# Qualitative analysis of a two-group SVIR epidemic model with random effect

**DOI:** 10.1186/s13662-021-03332-w

**Published:** 2021-03-19

**Authors:** Kaiyan Zhao, Shaojuan Ma

**Affiliations:** 1 School of Mathematics and Information Science, North Minzu University, Yinchuan, China; 2 The Key Laboratory of Intelligent Information and Big Data Processing of NingXia Province, Yinchuan, China

**Keywords:** Stochastic SVIR model, Brownian motion, *Itô*’s formula, Global positive solution, Properties of the solution, Asymptotic behavior

## Abstract

In this paper, we investigate the dynamical behavior of a two-group SVIR epidemic model with random effect. Firstly, the two-group SVIR epidemic model with random perturbation of natural death rate is established. The existence and uniqueness of positive solution are proved by using stopping time theory and the Lyapunov analysis method. Secondly, a property of the system solution is obtained by using the law of strong numbers and the continuous local martingale. Finally, a new combination of Lyapunov functions is applied. The solution of the model we obtained is oscillating around a steady state if the basic reproduction number is less than one, which is the disease-free equilibrium of the corresponding deterministic model. A numerical simulation is presented to verify our theoretical results.

## Introduction

The epidemic is one of the most important diseases which are caused by various pathogens and are harmful to the health of humans. It can be spread from person to person, from person to animal, or from animal to animal. Infectious diseases have always been the enemy of human survival and development. For a long time, human beings have been fighting with infectious diseases. For example, in 1988, a Malaysian village named Sungai Nipah was infected with an infectious disease called Nipah virus (Niv) [1]. The continued emergence of new major infectious diseases in the new century (such as SARS in 2003, A/H1H1 flu in 2009, MERS in 2012) and the epidemic of the novel coronavirus (COVID-19) have had a tremendous influence on the normal social life and people’s health. Therefore, the research on the transmission mechanism of infectious diseases has always been the focus of academia.

In the theory of epidemics, the main mathematical models used for a long time have been “chamber” models, which are still used widely and developed continuously. The classic “chamber” models, such as SIS, SIR, SIRS, etc., have been studied by many scholars [2–8]. Facts have proved that in order to control infectious diseases, prevention is one of the main means, and vaccination is the most effective way to prevent infectious diseases. Therefore, the model of an infectious disease with vaccinated SVIR is of great significance. In recent years, some scholars have proposed the SVIR model based on the original classical model and studied its dynamic behavior. Kribs-Zaleta and Velasco-Hernandez [9] added a chamber “V” to the SIS model, which represents that the population recovering from the disease return to the immune class rather than directly to the susceptible persons. Next, Lin and Takeuchi [10] built an SVIR model with a continuous vaccination strategy on this basis, the specific model is the following:

$$ \textstyle\begin{cases} \frac{dS}{dt}=\Lambda -\beta SI-\mu S-\theta S, \\ \frac{dV}{dt}=\theta S-\beta _{1}VI-\gamma _{1}V-\mu V, \\ \frac{dI}{dt}=\beta SI+\beta _{1}VI-\gamma I-\mu I, \\ \frac{dR}{dt}=\gamma _{1}V+\gamma I-\mu R. \end{cases} $$

Later, Ashrafu and Zou established a vaccine distribution model with vaccination priority [11]. However, the vaccination strategy model is not perfect. Biologically speaking, they ignored the situation where the vaccinees are not fully immune, that is, the vaccinees can still become infected again. In the real life, vaccination sometimes does not stop being infected completely, it just greatly reduces the possibility of being infected [12]. Therefore, it is necessary to consider the case of incomplete immunity in the model of infectious diseases. Reference [13] considered a basic SVIR epidemic model with age of vaccination and proposed that the model allows the vaccinated individuals to become susceptible again when the vaccine loses its protective properties over time. On this basis, the SVIR infectious disease model is studied, and the methods of combining *Itô*’s formula, the Lyapunov method, the LaSalle invariance principle, and a numerical simulation are introduced to analyze the global dynamics of the SVIR model [14]. In addition, Geng and Xu also applied the Mickens nonstandard finite difference format to the corresponding continuous model [15]. Many scholars have also considered age and time lag on the basis of this model [16, 17]. The optimal control of the basic deterministic SVIR model was analyzed by Liao and Yang [18]. Wang and Xu incorporated the nonlinear incidence into the SVIR epidemiological model of infected age and studied the local stability of each steady state of the model by analyzing the corresponding characteristic equation [19].

There have been many results which are about the dynamic behavior of the SVIR infectious disease model. In fact, we should consider the different connections and geographical locations of individuals when infectious diseases occur in life. Individuals in the population are not equally likely to be cured or be infected, and these inconsistencies among infectious disease populations are determined by a variety of factors. As a result, many scholars have begun to work on a multi-group epidemiological model of SVIR. Reference [20] used a multi-group infectious disease model to describe the spread of viruses in epidemiology. Toshikazu Kuniya and Yoshiki Muroya [21] proposed a global dynamic model of a multi-population SIS epidemic model with threshold parameter changes and analyzed its dynamic behavior. The global stability of the multi-group SVIR epidemic model based on the recently developed Lyapunov function and graph theory was described in [22]. In order to analyze the asymptotic stability at the two equilibrium points of a multi-group epidemiological model with variable separation of incidence and time lag, reference [23] proposed that the Liapunov function and the LaSalle invariance principles also apply to the multi-group epidemiological model.

The above research works are based on the deterministic SVIR epidemic model. In the real world, infectious disease models are naturally subject to random interference from the external environment. Therefore, it is more and more important to study the properties of infectious disease models under the influence of random factors. In recent years, some scholars have used the random driving force of Brownian motion as a random factor to join the infectious disease model to establish a random infectious disease model [24, 25]. The use of differential equation theory to study the stochastic epidemic model mainly draws dynamic conclusions such as global existence and uniqueness, stochastic stability, and progressive behavior of solutions [26, 27]. On this basis, the deterministic model and the corresponding random model are compared to analyze the changes that occur under the interference of random factors and then to grasp the trend of disease development, and provide important analysis for the study of the spread of diseases, disease prevention, and control departments [28].

However, it is well known that epidemic models are inevitably affected by the environmental noise. Due to the continuous fluctuation in the environment, the birth rates, death rates, transmission coefficient, and other parameters involved in the system should exhibit random fluctuation to a greater or lesser extent. However, because of the complexity of stochastic dynamics, there are not many results on the disturbance of mortality parameter. In this paper, based on the deterministic two-group SVIR infectious disease model in [29], as an extension of the deterministic system, we adopt a different approach to introduce random perturbation into it by replacing the natural mortality rate parameters, and a random two-group SVIR infectious disease model is established.

The model is developed in Sect. 2. The global positive solution is verified in Sect. 3, and the properties of the system solutions are studied in Sect. 4. The asymptotic behavior of the disease-free equilibrium is analyzed in Sect. 5. In Sect. 6, a numerical simulation is carried out to illustrate the results. The epidemiological ramifications of the results are presented in Sect. 7.

## Model building

The reference [29] investigates the consequences of vaccine implementation strategies for infectious diseases by a mathematical model. For an infectious disease, the degree of infection may vary from individual to individual. Reports show that individuals belonging to certain groups possess considerably higher risk of infection. In order to measure the outcome of the vaccination, the host is categorized into different groups by incorporating this phenomenon into vaccination strategies. And a mathematical model is proposed and analyzed to evaluate this measure.

$S^{r}(t)$, $S^{c}(t)$, $I^{r}(t)$, $I^{c}(t)$, $V^{r}(t)$, $V^{c}(t)$, $R^{r}(t)$, $R^{c}(t)$ represent the number of risky susceptible, critical susceptible, risky infective, critical infective, risky vaccinated, critical vaccinated, risky recovered, and critical recovered at moment *t*, respectively. Reference [29] considered the following deterministic model:

1 $$ \textstyle\begin{cases} \frac{dS^{r}(t)}{dt}=\Lambda ^{r}- ( \beta ^{r}_{r} \frac{I^{r}}{1+\alpha _{r}I^{r}}+\beta ^{r}_{c} \frac{I^{c}}{1+\alpha _{c}I^{c}} )S^{r}-(\mu +\theta ^{r})S^{r}, \\ \frac{dS^{c}(t)}{dt}=\Lambda ^{c}- ( \beta ^{c}_{r} \frac{I^{r}}{1+\alpha _{r}I^{r}}+\beta ^{c}_{c} \frac{I^{c}}{1+\alpha _{c}I^{c}} )S^{c}-(\mu +\theta ^{c})S^{c}, \\ \frac{dI^{r}(t)}{dt}= (\beta ^{r}_{r} \frac{I^{r}}{1+\alpha _{r}I^{r}}+\beta ^{r}_{c} \frac{I^{c}}{1+\alpha _{c}I^{c}} )S^{r}+ (\kappa ^{r}_{r} \frac{I^{r}}{1+\alpha _{r}I^{r}}+\kappa ^{r}_{c} \frac{I^{c}}{1+\alpha _{c}I^{c}} )V^{r}-(\mu +\nu ^{{r}}+\gamma ^{r})I^{r}, \\ \frac{dI^{c}(t)}{dt}= (\beta ^{c}_{r} \frac{I^{r}}{1+\alpha _{r}I^{r}}+\beta ^{c}_{c} \frac{I^{c}}{1+\alpha _{c}I^{c}} )S^{c}+ (\kappa ^{c}_{r} \frac{I^{r}}{1+\alpha _{r}I^{r}}+\kappa ^{c}_{c} \frac{I^{c}}{1+\alpha _{c}I^{c}} )V^{c}-(\mu +\nu ^{{c}}+\gamma ^{c})I^{c}, \\ \frac{dV^{r}(t)}{dt}=\theta ^{r}S^{r}- (\kappa ^{r}_{r} \frac{I^{r}}{1+\alpha _{r}I^{r}}+\kappa ^{r}_{c} \frac{I^{c}}{1+\alpha _{c}I^{c}} )V^{r}-\mu V^{r}, \\ \frac{dV^{c}(t)}{dt}=\theta ^{c}S^{c}- (\kappa ^{c}_{r} \frac{I^{r}}{1+\alpha _{r}I^{r}}+\kappa ^{c}_{c} \frac{I^{c}}{1+\alpha _{c}I^{c}} )V^{c}-\mu V^{c}, \\ \frac{dR^{r}(t)}{dt}=\gamma ^{r}I^{r}-\mu R^{r}, \\ \frac{dR^{c}(t)}{dt}=\gamma ^{c}I^{c}-\mu R^{c}, \end{cases} $$

where the parameters $\Lambda ^{i}$, $\beta ^{j}_{i}$, $\kappa ^{j}_{i}$, $\nu ^{i}$, *μ*, $\theta ^{i}$, $\gamma ^{i}$ are positive constants. Some notable features of the model are as follows: the influx of individuals into the susceptible is given by a constant $\Lambda ^{i}$; the natural death rates are assumed to be equal (denoted by constant *μ*), and individuals in $I^{i}(t)$ suffer additional death due to disease with rate constant $\nu ^{i}$; $\beta ^{j}_{i}$ and $\gamma ^{i}$ represent the disease transmission coefficient and the rate of recovery from infection respectively; $\kappa ^{j}_{i}$ represents the rate of contact between contacts and infected persons; $\theta ^{i}$ represents the contact rate of the risk group and the critical group ($i,j=r,c$). According to various contact methods of the crowd, the following can be obtained [29]:

$$ \begin{aligned} \beta ^{r}_{r}\gg \beta ^{c}_{c}, \quad\quad \beta ^{r}_{r}\gg \kappa ^{r}_{r}, \quad\quad \beta ^{c}_{c} \gg \kappa ^{c}_{c}, \end{aligned} $$

where $\beta ^{r}_{r}\gg \beta ^{c}_{c}$ means that the contact rate between risky susceptible persons and risky infected persons is much higher than that between critical susceptible persons and critical infected persons; $\beta ^{r}_{r}\gg \kappa ^{r}_{r}$ means that the contact rate between risky susceptible persons and risky infected persons is much higher than that between risky vaccinated and risky infected persons; $\beta ^{c}_{c}\gg \kappa ^{c}_{c}$ means that the contact rate between critical susceptible and critical infected persons is much higher than that between critical vaccinated persons and critical infected persons.

In model (1), except for $\frac{dR^{r}}{dt}$ and $\frac{dR^{c}}{dt}$, all equations are independent of $R^{r}(t)$ and $R^{c}(t)$, so the following simplified model can be obtained [29]:

2 $$ \textstyle\begin{cases} \frac{dS^{r}(t)}{dt}=\Lambda ^{r}- ( \beta ^{r}_{r} \frac{I^{r}}{1+\alpha _{r}I^{r}}+\beta ^{r}_{c} \frac{I^{c}}{1+\alpha _{c}I^{c}} )S^{r}-(\mu +\theta ^{r})S^{r}, \\ \frac{dS^{c}(t)}{dt}=\Lambda ^{c}- ( \beta ^{c}_{r} \frac{I^{r}}{1+\alpha _{r}I^{r}}+\beta ^{c}_{c} \frac{I^{c}}{1+\alpha _{c}I^{c}} )S^{c}-(\mu +\theta ^{c})S^{c}, \\ \frac{dI^{r}(t)}{dt}= (\beta ^{r}_{r} \frac{I^{r}}{1+\alpha _{r}I^{r}}+\beta ^{r}_{c} \frac{I^{c}}{1+\alpha _{c}I^{c}} )S^{r}+ (\kappa ^{r}_{r} \frac{I^{r}}{1+\alpha _{r}I^{r}}+\kappa ^{r}_{c} \frac{I^{c}}{1+\alpha _{c}I^{c}} )V^{r}-(\mu +\nu ^{{r}})I^{r}, \\ \frac{dI^{c}(t)}{dt}= (\beta ^{c}_{r} \frac{I^{r}}{1+\alpha _{r}I^{r}}+\beta ^{c}_{c} \frac{I^{c}}{1+\alpha _{c}I^{c}} )S^{c}+ (\kappa ^{c}_{r} \frac{I^{r}}{1+\alpha _{r}I^{r}}+\kappa ^{c}_{c} \frac{I^{c}}{1+\alpha _{c}I^{c}} )V^{c}-(\mu +\nu ^{{c}})I^{c}, \\ \frac{dV^{r}(t)}{dt}=\theta ^{r}S^{r}- (\kappa ^{r}_{r} \frac{I^{r}}{1+\alpha _{r}I^{r}}+\kappa ^{r}_{c} \frac{I^{c}}{1+\alpha _{c}I^{c}} )V^{r}-\mu V^{r}, \\ \frac{dV^{c}(t)}{dt}=\theta ^{c}S^{c}- (\kappa ^{c}_{r} \frac{I^{r}}{1+\alpha _{r}I^{r}}+\kappa ^{c}_{c} \frac{I^{c}}{1+\alpha _{c}I^{c}} )V^{c}-\mu V^{c}, \end{cases} $$

basic reproduction number:

$$ \begin{aligned} R_{0}=\rho \bigl(FV^{-1}\bigr), \end{aligned} $$

where

$$ \begin{aligned} F= \begin{vmatrix} \beta ^{r} _{r}\frac{\Lambda ^{r}}{\mu +\theta ^{r}}+\kappa ^{r}_{r} \frac{\theta ^{r}\Lambda ^{r}}{\mu ^{2}+\mu \theta ^{r}}& \beta ^{r} _{c} \frac{\Lambda ^{r}}{\mu +\theta ^{r}}+\kappa ^{r}_{c} \frac{\theta ^{r}\Lambda ^{r}}{\mu ^{2}+\mu \theta ^{r}} \\ \beta ^{c} _{r}\frac{\Lambda ^{c}}{\mu +\theta ^{c}}+\kappa ^{c}_{r} \frac{\theta ^{c}\Lambda ^{c}}{\mu ^{2}+\mu \theta ^{c}} & \beta ^{c} _{c} \frac{\Lambda ^{c}}{\mu +\theta ^{c}}+\kappa ^{c}_{c} \frac{\theta ^{c}\Lambda ^{c}}{\mu ^{2}+\mu \theta ^{c}} \end{vmatrix}, \quad\quad V= \begin{pmatrix} \mu +\nu ^{r} & 0 \\ 0 & \mu +\nu ^{{c}} \end{pmatrix} . \end{aligned} $$

When $R_{0}<1$, model (2) has the disease-free equilibrium $E_{0}= (\frac{\Lambda ^{r}}{\mu +\theta ^{r}} , \frac{\Lambda ^{c}}{\mu +\theta ^{c}} , 0 , 0 , \frac{\theta ^{r}\Lambda ^{r}}{\mu ^{2}+\mu \theta ^{r}} , \frac{\theta ^{c}\Lambda ^{c}}{\mu ^{2}+\mu \theta ^{c}} )$ which is globally asymptotically stable. If $R_{0}>1$, endemic equilibrium $E_{*}=(S^{r}_{*} , S^{c}_{*} , I^{r}_{*} , I^{c}_{*} , V^{r}_{*} , v^{c}_{*})$ is globally attractive. We mark

$$ \begin{aligned} N(t)={}&\Lambda ^{r}+\Lambda ^{c}-\mu S^{r}(t)-\mu S^{c}(t)-\bigl(\mu +\nu ^{{r}}\bigr)I^{r}(t)-\bigl( \mu +\nu ^{{c}} \bigr)I^{c}(t) \\ & {} -\mu V^{r}(t)-\mu V^{c} (t), \end{aligned} $$

then

$$ \begin{aligned} S^{r}(t)\geq 0, \quad\quad S^{c}(t)\geq 0, \quad\quad I^{r}(t)\geq 0, \quad\quad I^{c}(t)\geq 0, \quad\quad V^{r}(t) \geq 0, \quad\quad V^{c}(t)\geq 0, \end{aligned} $$

and

$$ \begin{aligned} \Gamma = \biggl\{ S^{r}(t)+S^{c}(t)+I^{r}(t)+I^{c}(t)+V^{r}(t)+V^{c}(t) \leq \frac{\Lambda ^{r}+\Lambda ^{c}}{\mu } \biggr\} . \end{aligned} $$

In fact, the parameters in the system are inevitably affected by random factors in the environment. Therefore this paper considers the situation where the natural death rate parameter in system (2) is disturbed. Here we assume that stochastic perturbations are of a white noise type which are directly proportional to $S^{r}(t)$, $S^{c}(t)$, $I^{r}(t)$, $I^{c}(t)$, $V^{r}(t)$, $V^{c}(t)$, influenced on the $\dot{S^{r}(t)}$, $\dot{S^{c}(t)}$, $\dot{I^{r}(t)}$, $\dot{I^{c}(t)}$, $\dot{V^{r}(t)}$, $\dot{V^{c}(t)}$, in model (2). By this way, model (2) will be deduced to the form:

3 $$ \textstyle\begin{cases} \frac{dS^{r}(t)}{dt}=\Lambda ^{r}- (\beta ^{r}_{r} \frac{I^{r}}{1+\alpha _{r}I^{r}}+\beta ^{r}_{c} \frac{I^{c}}{1+\alpha _{c}I^{c}} )S^{r}-(\mu +\theta ^{r})S^{r}+ \sigma _{1}S^{r}\,dB_{1}(t), \\ \frac{dS^{c}(t)}{dt}=\Lambda ^{c}- (\beta ^{c}_{r} \frac{I^{r}}{1+\alpha _{r}I^{r}}+\beta ^{c}_{c} \frac{I^{c}}{1+\alpha _{c}I^{c}} )S^{c}-(\mu +\theta ^{c})S^{c}+ \sigma _{2}S^{c}\,dB_{2}(t), \\ \frac{dI^{r}(t)}{dt}= (\beta ^{r}_{r} \frac{I^{r}}{1+\alpha _{r}I^{r}}+\beta ^{r}_{c} \frac{I^{c}}{1+\alpha _{c}I^{c}} )S^{r}+ (\kappa ^{r}_{r} \frac{I^{r}}{1+\alpha _{r}I^{r}}+\kappa ^{r}_{c} \frac{I^{c}}{1+\alpha _{c}I^{c}} )V^{r}-(\mu +\nu ^{{r}})I^{r} \\ \hphantom{\frac{dI^{r}(t)}{dt}}\quad{} +\sigma _{3}I^{r}\,dB_{3}(t), \\ \frac{dI^{c}(t)}{dt}= (\beta ^{c}_{r} \frac{I^{r}}{1+\alpha _{r}I^{r}}+\beta ^{c}_{c} \frac{I^{c}}{1+\alpha _{c}I^{c}} )S^{c}+ (\kappa ^{c}_{r} \frac{I^{r}}{1+\alpha _{r}I^{r}}+\kappa ^{c}_{c} \frac{I^{c}}{1+\alpha _{c}I^{c}} )V^{c}-(\mu +\nu ^{{c}})I^{c} \\ \hphantom{\frac{dI^{c}(t)}{dt}}\quad{} +\sigma _{4}I^{c}\,dB_{4}(t), \\ \frac{dV^{r}(t)}{dt}=\theta ^{r}S^{r}- (\kappa ^{r}_{r} \frac{I^{r}}{1+\alpha _{r}I^{r}}+\kappa ^{r}_{c} \frac{I^{c}}{1+\alpha _{c}I^{c}} )V^{r}-\mu V^{r}+\sigma _{5}V^{r}\,dB_{5}(t), \\ \frac{dV^{c}(t)}{dt}=\theta ^{c}S^{c}- (\kappa ^{c}_{r} \frac{I^{r}}{1+\alpha _{r}I^{r}}+\kappa ^{c}_{c} \frac{I^{c}}{1+\alpha _{c}I^{c}} )V^{c}-\mu V^{c}+\sigma _{6}V^{c}\,dB_{5}(t), \end{cases} $$

where $B_{i}(t)$ are independent standard Brownian motions and $\sigma _{i}^{2}\geq 0$ represent the intensities of $B_{i}(t)$, $i=1,2,3,\ldots,6$.

Obviously, $E_{0}= (\frac{\Lambda ^{r}}{\mu +\theta ^{r}}, \frac{\Lambda ^{c}}{\mu +\theta ^{c}},0,0, \frac{\theta ^{r}\Lambda ^{r}}{\mu ^{2}+\mu \theta ^{r}}, \frac{\theta ^{c}\Lambda ^{c}}{\mu ^{2}+\mu \theta ^{c}} )$ is the disease-free equilibrium point of the random system (3).

## Global positive solution

In order to investigate the dynamical behavior, the first thing we are concerned with is whether the solution is global existence. Moreover, for a model of population dynamics, whether the value of the solution is nonnegative is also considered. Hence in this section we show that the solution of system (3) is global and nonnegative. As we know, in order to make a stochastic differential equation have a unique global (i.e. no explosion in a finite time) solution for any given initial value, the coefficients of the equation are generally required to satisfy the linear growth condition and the local Lipschitz condition (cf. [30]). However, the coefficients of model (3) do not satisfy the linear growth condition, though they are locally Lipschitz continuous, so the solution of model (3) may explode at a finite time (cf. [30]). In this section, using the Lyapunov analysis method (mentioned in [30]), we show that the solution of model (3) is positive and global.

### Theorem 3.1

*For any given initial value* $\{S^{r}(0), S^{c}(0), I^{r}(0), I^{c}(0), V^{r}(0), V^{c}(0)\}\in \mathbb{R}^{6}_{+}$, *there is a unique positive solution* $\{S^{r}(t), S^{c}(t), I^{r}(t), I^{c}(t), V^{r}(t), V^{c}(t)\}$ *of model* (3) *on* $t\geq 0$ *and the solution will remain in* $\mathbb{R}^{6}_{+}$ *with probability one*, *namely* $\{S^{r}(t), S^{c}(t), I^{r}(t), I^{c}(t), V^{r}(t), V^{c}(t)\}\in \mathbb{R}^{6}_{+}$ *for all* $t\geq 0$ *almost surely*.

### Proof

Since the coefficients of the equation are locally Lipschitz continuous, for any given initial value $\{S^{r}(0) , S^{c}(0) , I^{r}(0) , I^{c}(0) , V^{r}(0) , V^{c}(0)\}\in \mathbb{R}^{6}_{+}$, there is a unique local solution $\{S^{r}(t) , S^{c}(t) , I^{r}(t) , I^{c}(t) , V^{r}(t) , V^{c}(t)\}$ on $t\in [0,\tau _{e})$, where $\tau _{e}$ is the explosion time. To show that this solution is global, we need to show that $\tau _{e}=\infty $ a.s. We prove that $S^{r}(t)$, $S^{c}(t)$, $I^{r}(t)$, $I^{c}(t)$, $V^{r}(t)$, $V^{c}(t)$ do not explode to infinity in a finite time. Set $k_{0}\geq 1$ to be sufficiently large for $S^{r}(0)\in [\frac{1}{k_{0}},k_{0} ]$, $S^{c}(0)\in [\frac{1}{k_{0}},k_{0} ]$, $I^{r}(0)\in [\frac{1}{k_{0}},k_{0} ]$, $I^{c}(0)\in [\frac{1}{k_{0}},k_{0} ]$, $V^{r}(0)\in [\frac{1}{k_{0}},k_{0} ]$, $V^{c}(0)\in [\frac{1}{k_{0}},k_{0} ]$. For each integer $k>k_{0}$, define the stopping time

$$ \begin{aligned} \tau _{k}={}&\inf \biggl\{ t\in [0,\tau _{e}):S^{r}(t)\notin \biggl( \frac{1}{k},k \biggr) \text{ or } S^{c}(t)\notin \biggl(\frac{1}{k},k \biggr)\text{ or } I^{r}(t)\notin \biggl(\frac{1}{k},k \biggr) \\ &\text{or } I^{c}(t)\notin \biggl(\frac{1}{k},k \biggr)\text{ or } V^{r}(t) \notin \biggl(\frac{1}{k},k \biggr)\text{ or } V^{c}(t)\notin \biggl(\frac{1}{k},k \biggr) \biggr\} , \end{aligned} $$

where throughout this paper we set $\inf \emptyset =\infty$ (∅ denotes the empty set). Obviously, $\tau _{k}$ is increasing as $k\rightarrow \infty $. Set $\tau _{\infty }=\lim_{k\rightarrow \infty }\tau _{k}$, therefore $\tau _{\infty }<\tau _{e}$ a.s. If $\tau _{\infty }<\infty $ a.s. is true, then $\tau _{e}=\infty $ a.s. and $\{S^{r}(t) , S^{c}(t) , I^{r}(t) , I^{c}(t) , V^{r}(t) , V^{c}(t)\}\in \mathbb{R}^{6}_{+}$ a.s. for $t\geq 0$. In other words, to complete the proof, it is required to show that $\tau _{\infty }=\infty $ a.s. If this statement is false, then there exist a pair of constants $T>0$ and $\epsilon \in (0,1)$ such that

$$ \begin{aligned} P\{\tau _{\infty }\leq T\}>\epsilon , \end{aligned} $$

thus there is an integer $k_{1}\geq k_{0}$ such that

4 $$ \begin{aligned} P\{\tau _{k}\leq T\}\geq \epsilon \quad \forall k\geq k_{1}. \end{aligned} $$

Let us define a $C^{2}$-function $V:\mathbb{R}^{6}_{+}\rightarrow \mathbb{R}_{+}$ as follows:

$$ \begin{aligned} V\bigl(S^{r},S^{c},I^{r},I^{c},V^{r},V^{c} \bigr)={}&\bigl(S^{r}-1-\ln S^{r}\bigr)+\bigl(S^{c}-1- \ln S^{c}\bigr)+\bigl(I^{r}-1-\ln I^{r}\bigr) \\ &{}+\bigl(I^{c}-1-\ln I^{c}\bigr)+\bigl(V^{r}-1-\ln V^{r}\bigr)+\bigl(V^{c}-1-\ln V^{c}\bigr). \end{aligned} $$

The nonnegativity of this function can be seen from

$$ \begin{aligned} u-1-\ln u\geq 0 \quad \forall u>0. \end{aligned} $$

Let $k\geq k_{0}$ and $T>0$ be arbitrary. Applying *Itô*’s formula, we obtain

$$ \begin{aligned} dV\bigl(S^{r},S^{c},I^{r},I^{c},V^{r},V^{c} \bigr)={}&LV\bigl(S^{r},S^{c},I^{r},I^{c},V^{r},V^{c} \bigr)\,dt+ \sigma _{1}\bigl(S^{r}-1\bigr) \,dB_{1}(t) \\ &{}+\sigma _{2}\bigl(S^{c}-1\bigr)dB_{2}(t)+\sigma _{3}\bigl(I^{r}-1\bigr) \,dB_{3}(t)+\sigma _{4}\bigl(I^{c}-1\bigr) \,dB_{4}(t) \\ &{}+ \sigma _{5}\bigl(V^{r}-1\bigr)dB_{5}(t)+\sigma _{6}\bigl(V^{c}-1\bigr) \,dB_{6}(t), \end{aligned} $$

where $LV:\mathbb{R}^{6}_{+}\rightarrow \mathbb{R}_{+}$ is defined by

$$ \begin{aligned} &LV\bigl(S^{r},S^{c},I^{r},I^{c},V^{r},V^{c} \bigr) \\ &\quad = \biggl(1-\frac{1}{S^{r}} \biggr) \biggl[\Lambda ^{r}- \biggl(\beta ^{r}_{r}\frac{I^{r}}{1+\alpha _{r}I^{r}}+ \beta ^{r}_{c}\frac{I^{c}}{1+\alpha _{c}I^{c}} \biggr)S^{r} \\ &\quad \quad {} -\bigl(\mu +\theta ^{r}\bigr)S^{r} \biggr]+ \biggl(1- \frac{1}{S^{c}} \biggr) \biggl[ \Lambda ^{c}- \biggl(\beta ^{c}_{r}\frac{I^{r}}{1+\alpha _{r}I^{r}}+ \beta ^{c}_{c} \frac{I^{c}}{1+\alpha _{c}I^{c}} \biggr)S^{c}-\bigl(\mu + \theta ^{c} \bigr)S^{c} \biggr] \\ &\quad \quad {} + \biggl(1-\frac{1}{I^{r}} \biggr) \biggl[ \biggl(\beta ^{r}_{r} \frac{I^{r}}{1+\alpha _{r}I^{r}}+\beta ^{r}_{c} \frac{I^{c}}{1+\alpha _{c}I^{c}} \biggr)S^{r}+ \biggl(\kappa ^{r}_{r} \frac{I^{r}}{1+\alpha _{r}I^{r}}+\kappa ^{r}_{c} \frac{I^{c}}{1+\alpha _{c}I^{c}} \biggr)V^{r} \\ &\quad \quad {} -\bigl(\mu +\nu ^{r}\bigr)I^{r} \biggr]+ \frac{1}{2}\sigma ^{2}_{1}+\frac{1}{2} \sigma ^{2}_{2}+\frac{1}{2}\sigma ^{2}_{3}+ \biggl(1-\frac{1}{I^{c}} \biggr) \biggl[ \biggl(\beta ^{c}_{r} \frac{I^{r}}{1+\alpha _{r}I^{r}}+\beta ^{c}_{c} \frac{I^{c}}{1+\alpha _{c}I^{c}} \biggr)S^{c} \\ &\quad \quad {} + \biggl(\kappa ^{c}_{r}\frac{I^{r}}{1+\alpha _{r}I^{r}}+\kappa ^{c}_{c} \frac{I^{c}}{1+\alpha _{c}I^{c}} \biggr)V^{c}-\bigl(\mu +\nu ^{c}\bigr)I^{c} \biggr]+ \frac{1}{2}\sigma ^{2}_{4}+\frac{1}{2}\sigma ^{2}_{5}+ \frac{1}{2} \sigma ^{2}_{6} \\ &\quad \quad {} + \biggl(1-\frac{1}{V^{r}} \biggr) \biggl[\theta ^{r}S^{r}- \biggl(\kappa ^{r}_{r} \frac{I^{r}}{1+\alpha _{r}I^{r}}+\kappa ^{r}_{c} \frac{I^{c}}{1+\alpha _{c}I^{c}} \biggr)V^{r}-\mu V^{r} \biggr] \\ &\quad \quad {} + \biggl(1-\frac{1}{V^{c}} \biggr) \biggl[\theta ^{c}S^{c}- \biggl(\kappa ^{c}_{r} \frac{I^{r}}{1+\alpha _{r}I^{r}}+\kappa ^{c}_{c} \frac{I^{c}}{1+\alpha _{c}I^{c}} \biggr)V^{c}-\mu V^{c} \biggr]. \end{aligned} $$

Therefore

$$ \begin{aligned} LV\bigl(S^{r},S^{c},I^{r},I^{c},V^{r},V^{c} \bigr)\leq{}& \biggl\{ \Lambda ^{r}+ \frac{\beta ^{r}_{r}I^{r}}{1+\alpha _{r}I^{r}}+ \frac{\beta ^{r}_{c}I^{c}}{1+\alpha _{c}I^{c}}+\bigl(\mu +\theta ^{r}\bigr)+ \Lambda ^{c}+ \frac{\beta ^{c}_{r}I^{r}}{1+\alpha _{r}I^{r}} \\ & {} +\frac{\beta ^{c}_{c}I^{c}}{1+\alpha _{c}I^{c}}+\bigl(\mu +\theta ^{c}\bigr)+\bigl( \mu +\nu ^{r}\bigr)+\bigl(\mu +\nu ^{c}\bigr)+\theta ^{r}S^{r} \\ & {} +\theta ^{c}S^{c}+\frac{\kappa ^{r}_{r}I^{r}}{1+\alpha _{r}I^{r}}+ \frac{\kappa ^{r}_{c}I^{c}}{1+\alpha _{c}I^{c}}+\mu + \frac{\kappa ^{c}_{r}I^{r}}{1+\alpha _{r}I^{r}}+ \frac{\kappa ^{c}_{c}I^{c}}{1+\alpha _{c}I^{c}} \\ & {} +\mu +\frac{1}{2}\bigl(\sigma ^{2}_{1}+\sigma ^{2}_{2}+\sigma ^{2}_{3}+ \sigma ^{2}_{4}+\sigma ^{2}_{5}+\sigma ^{2}_{6}\bigr) \biggr\} \,dt. \end{aligned} $$

By simplifying $\frac{x}{1+\alpha _{i}x}$ ($i=c,r$) to *x* then

$$ \begin{aligned} LV\bigl(S^{r},S^{c},I^{r},I^{c},V^{r},V^{c} \bigr)\leq{}& \Lambda ^{r}+\Lambda ^{c}+\bigl( \beta ^{r}_{r}+\beta ^{r}_{c}+\beta ^{c}_{r}+\beta ^{c}_{c}\bigr)+\bigl( \theta ^{r}+ \theta ^{c}\bigr)+\bigl(\nu ^{r}+\nu ^{c}\bigr)+6\mu \\ & {} +\bigl(\kappa ^{r}_{r}+\kappa ^{r}_{c}+ \kappa ^{c}_{r}+\kappa ^{c}_{c}\bigr)+ \frac{1}{2}\bigl(\sigma ^{2}_{1}+\sigma ^{2}_{2}+\sigma ^{2}_{3}+\sigma ^{2}_{4}+ \sigma ^{2}_{5}+\sigma ^{2}_{6}\bigr) \\ :={}&K, \end{aligned} $$

where $K\in N^{+}$, so

5 $$ \begin{aligned} dV\bigl(S^{r},S^{c},I^{r},I^{c},V^{r},V^{c} \bigr)\leq{}& K\,dt+\bigl[\sigma _{1}\bigl(S^{r}-1\bigr) \,dB_{1}(t)+ \sigma _{2}\bigl(S^{c}-1\bigr) \,dB_{2}(t) \\ & {} +\sigma _{3}\bigl(I^{r}-1\bigr)\,dB_{3}(t)+ \sigma _{4}\bigl(I^{c}-1\bigr)\,dB_{4}(t) \\ & {} +\sigma _{5}\bigl(V^{r}-1\bigr)\,dB_{5}(t)+ \sigma _{6}\bigl(V^{c}-1\bigr)\,dB_{6}(t)\bigr]. \end{aligned} $$

We can now integrate both sides of (5) from to $\tau _{k}\wedge T$ and then take the expectations

$$ \begin{aligned} &EV\{S^{r}(\tau _{k}\wedge T),S^{c}(\tau _{k}\wedge T),I^{r}(\tau _{k} \wedge T),I^{c}(\tau _{k}\wedge T),V^{r}(\tau _{k}\wedge T),V^{c}( \tau _{k}\wedge T) \\ &\quad \leq V\bigl\{ S^{r}(0),S^{c}(0),I^{r}(0),I^{c}(0),V^{r}(0),V^{c}(0) \bigr\} +KE( \tau _{k}\wedge T), \end{aligned} $$

so

6 $$ \begin{aligned} &EV\{S^{r}(\tau _{k}\wedge T),S^{c}(\tau _{k}\wedge T),I^{r}(\tau _{k} \wedge T),I^{c}(\tau _{k}\wedge T),V^{r}(\tau _{k}\wedge T),V^{c}( \tau _{k}\wedge T) \\ &\quad \leq V\bigl\{ S^{r}(0),S^{c}(0),I^{r}(0),I^{c}(0),V^{r}(0),V^{c}(0) \bigr\} +KT. \end{aligned} $$

Let $\Omega _{k}=\{\tau _{k}\leq T\}$ for $k\geq k_{1}$ and, by (4), $P(\Omega _{k})\geq \epsilon $. Note that, for every $\omega \in \Omega _{k}$, there is $S^{r}(\tau _{k},\omega )$ or $S^{c}(\tau _{k},\omega )$ or $I^{r}(\tau _{k},\omega )$ or $I^{c}(\tau _{k},\omega )$ or $V^{r}(\tau _{k},\omega )$ or $V^{c}(\tau _{k},\omega )$ equals either *k* or $\frac{1}{k}$, and therefore $V\{S^{r}(\tau _{k},\omega ), S^{c}(\tau _{k},\omega ), I^{r}(\tau _{k},\omega ), I^{c}(\tau _{k}, \omega ),V^{r}(\tau _{k},\omega ),V^{c}(\tau _{k},\omega )\}$ is no less than either

$$ \begin{aligned} k-1-\ln k\quad \text{or}\quad \frac{1}{k}-1- \ln \frac{1}{k}=\frac{1}{k}-1+ \ln k. \end{aligned} $$

Hence

$$ \begin{aligned} &\bigl\{ S^{r}(\tau _{k},\omega ),S^{c}(\tau _{k},\omega ),I^{r}(\tau _{k}, \omega ),I^{c}(\tau _{k},\omega ),V^{r}(\tau _{k},\omega ),V^{c}( \tau _{k},\omega )\bigr\} \\ &\quad \geq [k-1-\ln k]\wedge \biggl[\frac{1}{k}-1+\ln k \biggr]. \end{aligned} $$

It then follows from (6) that

$$ \begin{aligned} &V\bigl\{ S^{r}(0),S^{c}(0),I^{r}(0),I^{c}(0),V^{r}(0),V^{c}(0) \bigr\} +KT \\ &\quad \geq E\bigl[1_{\Omega _{k}}(\omega )V\bigl\{ S^{r}(\tau _{k},\omega ),S^{c}( \tau _{k},\omega ),I^{r}(\tau _{k},\omega ),I^{c}(\tau _{k},\omega ),V^{r}( \tau _{k},\omega ),V^{c}(\tau _{k},\omega )\bigr\} \bigr] \\ &\quad \geq \epsilon [k-1-\ln k]\wedge \biggl[\frac{1}{k}-1+\ln k \biggr], \end{aligned} $$

where $1_{\Omega _{k}}$ is the indicator function of $\Omega _{k}$, letting $k\rightarrow \infty $, we have that

$$ \begin{aligned} \infty > V\bigl\{ S^{r}(0),S^{c}(0),I^{r}(0),I^{c}(0),V^{r}(0),V^{c}(0) \bigr\} +KT= \infty \end{aligned} $$

is a contradiction, then we must have

$$ \begin{aligned} \tau _{\infty }=\infty . \end{aligned} $$

Therefore, it implies $S^{r}(t)$, $S^{c}(t)$, $I^{r}(t)$, $I^{c}(t)$, $V^{r}(t)$, $V^{c}(t)$ will not explode in a finite time with probability one. That is, system (3) has a unique global positive solution. □

It can be seen from Theorem 3.1 that no matter how big the noise intensity $\sigma _{1}$, $\sigma _{2}$, $\sigma _{3}$, $\sigma _{4}$, $\sigma _{5}$, $\sigma _{6}$ is there must be a unique global positive solution for any initial value given by the random model (3).

## Properties of the solution

In this chapter, we use the law of strong numbers and the properties of continuous local martingales to study the properties of system solutions, provide some theoretical support for the future work of the random control of the random model.

### Theorem 4.1

*Assuming that* $\{S^{r}(t),S^{c}(t),I^{r}(t),I^{c}(t),V^{r}(t),V^{c}(t)\}$ *is a positive solution of system* (3), *we have*

$$ \limsup_{t\rightarrow \infty }\bigl\{ S^{r}(t)+S^{c}(t)+I^{r}(t)+I^{c}(t)+V^{r}(t)+V^{c}(t) \bigr\} < \infty \quad \textit{a.s.} $$

*and*

$$ \begin{aligned} &\limsup_{t\rightarrow \infty }\bigl\langle S^{r}(t)\bigr\rangle \leq \frac{\Lambda ^{r}+\Lambda ^{c}}{\mu },\quad \quad \limsup _{t \rightarrow \infty }\bigl\langle S^{c}(t)\bigr\rangle \leq \frac{\Lambda ^{r}+\Lambda ^{c}}{\mu } \quad \textit{a.s.}, \\ &\limsup_{t\rightarrow \infty }\bigl\langle I^{r}(t)\bigr\rangle \leq \frac{\Lambda ^{r}+\Lambda ^{c}}{\mu +\nu ^{r}},\quad \quad \limsup_{t\rightarrow \infty }\bigl\langle I^{c}(t)\bigr\rangle \leq \frac{\Lambda ^{r}+\Lambda ^{c}}{\mu +\nu ^{c}} \quad \textit{a.s.}, \\ &\limsup_{t\rightarrow \infty }\bigl\langle V^{r}(t)\bigr\rangle \leq \frac{\Lambda ^{r}+\Lambda ^{c}}{\mu },\quad \quad \limsup_{t \rightarrow \infty }\bigl\langle V^{c}(t)\bigr\rangle \leq \frac{\Lambda ^{r}+\Lambda ^{c}}{\mu } \quad \textit{a.s.} \end{aligned} $$

*and*

$$ \begin{aligned} &\lim_{t\rightarrow \infty }\frac{M_{i}(t)}{t}=0 \quad (i=1,2,\ldots,6), \end{aligned} $$

*where* $M_{1}(t)=\sigma _{1}\int _{0}^{t}S^{r}(s)\,dB_{1}(s)$, $M_{2}(t)=\sigma _{2}\int _{0}^{t}S^{c}(s)\,dB_{2}(s)$, $M_{3}(t)=\sigma _{3}\int _{0}^{t}I^{r}(s)\,dB_{3}(s)$, $M_{4}(t)=\sigma _{4}\int _{0}^{t}I^{c}(s)\,dB_{1}(s)$, $M_{5} (t) =\sigma _{5}\int _{0}^{t}V^{r}(s)\,dB_{5}(s)$, $M_{6}(t)=\sigma _{6}\int _{0}^{t}V^{c}(s)\,dB_{6}(s)$.

### Proof

Order $N=S^{r}+S^{c}+I^{r}+I^{c}+V^{r}+V^{c}$, then *N* satisfies (3)

$$ \begin{aligned} dN(t)={}&\Lambda ^{r}+\Lambda ^{c}-\mu N-\nu ^{r}I^{r}-\nu ^{c}I^{c}+ \sigma _{1}S^{r}\,dB_{1}(t)++\sigma _{2}S^{c}\,dB_{2}(t) \\ & {} +\sigma _{3}I^{r}\,dB_{3}(t)+\sigma _{4}I^{c}\,dB_{4}(t)+\sigma _{5}V^{r} \,dB_{5}(t)+ \sigma _{6}V^{c}\,dB_{6}(t). \end{aligned} $$

Therefore

$$ \begin{aligned} N(t)={}& N(0)e^{-\mu t}+ \biggl( \frac{\Lambda ^{r}+\Lambda ^{c}}{\mu } \biggr) \bigl(1-e^{- \mu t}\bigr)-\nu ^{r} \int _{0}^{t}e^{\mu (s-t)}I^{r}(s)\,ds \\ & {} -\nu ^{c} \int _{0}^{t}e^{\mu (s-t)}I^{c}(s) \,ds+M(t),\end{aligned} $$

where $M(t)=\sigma _{1}\int _{0}^{t}e^{\mu (s-t)}S^{r}(s)\,dB_{1}(s) +\sigma _{2}\int _{0}^{t}e^{\mu (s-t)}S^{c}(s)\,dB_{2}(s) +\sigma _{3}\int _{0}^{t}e^{\mu (s-t)}I^{r}(s)\,dB_{3}(s) +\sigma _{4}\int _{0}^{t}e^{\mu (s-t)}I^{c}(s)\,dB_{4}(s) +\sigma _{5}\int _{0}^{t}e^{\mu (s-t)}V^{r}(s)\,dB_{5}(s) +\sigma _{6}\int _{0}^{t}e^{\mu (s-t)}V^{c}(s)\,dB_{6}(s)$ is a continuous local martingale that satisfies $M(0)=0$, remember

$$ \begin{aligned} X(t)=X(0)+A(t)-U(t)+M(t), \end{aligned} $$

where $X(0)=N(0)$, $A(t)= (\frac{\Lambda ^{r}+\Lambda ^{c}}{\mu } )(1-e^{-\mu t})$, $U(t)=N(0)(1-e^{-\mu t})$. Therefore $N(t)\leq X(t)$. Obviously $A(t)$ and $U(t)$ are continuous adaptive incremental processes that satisfy $A(0)=U(0)=0$. From Lemma 2.1 [31], $\lim_{t\rightarrow \infty }X(t)<\infty $ a.s. $\limsup_{t\rightarrow \infty }N(t)<\infty $ a.s. From this it can be seen that

$$ \begin{aligned} \limsup_{t\rightarrow \infty } \frac{\langle M_{1},M_{1}\rangle }{t}= \limsup_{t\rightarrow \infty }\frac{\sigma ^{2}_{1}}{t} \int _{0}^{t}\bigl(S^{r} \bigr)^{2}(s)\,ds\leq \sigma ^{2}_{1}\sup _{t\geq 0}\bigl(S^{r}\bigr)^{2}(t)< \infty , \end{aligned} $$

and then by the law of strong numbers

7 $$ \begin{aligned} \lim_{t\rightarrow \infty }\frac{M_{1}(t)}{t}=0\quad \textit{a.s.} \end{aligned} $$

The same is true

8 $$ \begin{aligned} \lim_{t\rightarrow \infty }\frac{M_{i}(t)}{t}=0\quad \textit{a.s. } (i=2,3,\ldots,6). \end{aligned} $$

Obtained by system (3)

$$ \begin{aligned} dN(t)={}&\Lambda ^{r}+\Lambda ^{c}-\mu S^{r}-\mu S^{c}-\bigl(\mu +\nu ^{r}\bigr)I^{r}-\bigl( \mu +\nu ^{c} \bigr)I^{c}-\mu V^{r}-\mu V^{c}+\sigma _{1}S^{r}\,dB_{1}(t) \\ & {} +\sigma _{2}S^{c}\,dB_{2}(t)+\sigma _{3}I^{r}\,dB_{3}(t)+\sigma _{4}I^{c} \,dB_{4}(t)+ \sigma _{5}V^{r}\,dB_{5}(t)+ \sigma _{6}V^{c}\,dB_{6}(t). \end{aligned} $$

Therefore

$$ \begin{aligned} \bigl\langle S^{r}(t)\bigr\rangle ={}& \frac{\Lambda ^{r}+\Lambda ^{c}}{\mu }- \bigl\langle S^{c}(t)\bigr\rangle - \frac{\mu +\nu ^{r}}{\mu }\bigl\langle I^{r}(t) \bigr\rangle -\frac{\mu +\nu ^{c}}{\mu } \bigl\langle I^{c}(t)\bigr\rangle -\bigl\langle V^{r}(t) \bigr\rangle -\bigl\langle V^{c}(t)\bigr\rangle \\ & {} +\frac{1}{\mu } \biggl[ \frac{\int _{0}^{t}N(s)\,ds+\sigma _{1}\int _{0}^{t}S^{r}\,dB_{1}(t)+\sigma _{2}\int _{0}^{t}S^{c}\,dB_{2}(t)+\sigma _{3}\int _{0}^{t}I^{r}\,dB_{3}(t)}{t} \biggr] \\ & {} +\frac{1}{\mu } \biggl[ \frac{\sigma _{4}\int _{0}^{t}I^{c}\,dB_{4}(t)+\sigma _{5}\int _{0}^{t}V^{r}\,dB_{5}(t)+\sigma _{6}\int _{0}^{t}V^{c}\,dB_{6}(t)}{t} \biggr], \end{aligned} $$

so

$$ \begin{aligned} \bigl\langle S^{r}(t)\bigr\rangle ={}& \frac{\Lambda ^{r}+\Lambda ^{c}}{\mu }- \bigl\langle S^{c}(t)\bigr\rangle - \frac{\mu +\nu ^{r}}{\mu }\bigl\langle I^{r}(t) \bigr\rangle -\frac{\mu +\nu ^{c}}{\mu } \bigl\langle I^{c}(t)\bigr\rangle \\ & {} -\bigl\langle V^{r}(t)\bigr\rangle -\bigl\langle V^{c}(t)\bigr\rangle + \frac{\varphi (t)}{t}, \end{aligned} $$

where

$$ \begin{aligned} \varphi (t)=\frac{1}{\mu }\bigl\{ \bigl[N(t)-N(0) \bigr]+M_{1}(t)+M_{2}(t)+M_{3}(t)+M_{4}(t)+M_{5}(t)+M_{6}(t) \bigr\} . \end{aligned} $$

We know $\frac{\varphi (t)}{t}\rightarrow 0 $ ($t\rightarrow \infty $) from (7–8), then

$$ \begin{aligned} \limsup_{t\rightarrow \infty }\bigl\langle S^{r}(t)\bigr\rangle \leq \frac{\Lambda ^{r}+\Lambda ^{c}}{\mu }. \end{aligned} $$

By the same token

$$ \begin{aligned} &\limsup_{t\rightarrow \infty }\bigl\langle S^{c}(t)\bigr\rangle \leq \frac{\Lambda ^{r}+\Lambda ^{c}}{\mu },\quad\quad \limsup _{t \rightarrow \infty }\bigl\langle I^{r}(t)\bigr\rangle \leq \frac{\Lambda ^{r}+\Lambda ^{c}}{\mu +\nu ^{r}},\quad\quad \limsup_{t\rightarrow \infty }\bigl\langle I^{c}(t)\bigr\rangle \leq \frac{\Lambda ^{r}+\Lambda ^{c}}{\mu +\nu ^{c}}, \\ &\limsup_{t\rightarrow \infty }\bigl\langle V^{r}(t)\bigr\rangle \leq \frac{\Lambda ^{r}+\Lambda ^{c}}{\mu },\quad\quad \limsup_{t \rightarrow \infty }\bigl\langle V^{c}(t)\bigr\rangle \leq \frac{\Lambda ^{r}+\Lambda ^{c}}{\mu }. \end{aligned} $$

 □

## Asymptotic behavior of the disease-free equilibrium

Theorem 3.1 shows that the stochastically perturbed system (3) will remain to have a global positive solution. In the sequel we therefore only need to consider how the solution varies in ${R}^{6}_{+}$. Since model (3) does not have an explicit solution, the study of asymptotic behavior is essential. The asymptotic property of model (3) is given by the following theorem.

### Theorem 5.1

*If* $R_{0}<1$ *and the following conditions are satisfied*:

$$ \begin{aligned} &\sigma _{1}^{2}< \frac{1}{1+p} \biggl( \frac{p\mu (\mu +\theta ^{r})}{\mu +2\theta ^{r}}-\bigl(2\mu +\nu ^{r}\bigr) \biggr), \quad\quad \sigma _{2}^{2}< \frac{1}{1+q} \biggl( \frac{q\mu (\mu +\theta ^{c})}{\mu +2\theta ^{c}}-\bigl(2\mu +\nu ^{c}\bigr) \biggr), \\ &\sigma _{3}^{2}< \bigl(\mu +\nu ^{r}\bigr), \quad\quad \sigma _{4}^{2}< \bigl(\mu +\nu ^{c} \bigr), \\ &\sigma _{5}^{2}< \frac{\theta ^{r}}{p\mu +\theta ^{r}} \biggl( \frac{p\mu ^{3}}{2\theta ^{r}(\theta ^{r}+\mu )}-\bigl(2\mu +\nu ^{r}\bigr) \biggr), \\ &\sigma _{6}^{2}< \frac{\theta ^{c}}{q\mu +\theta ^{c}} \biggl( \frac{q\mu ^{3}}{2\theta ^{c}(\theta ^{c}+\mu )}- \bigl(2\mu +\nu ^{c}\bigr) \biggr), \end{aligned} $$

*then for any given initial value* $\{S^{r}(0),S^{c}(0),I^{r}(0),I^{c}(0),V^{r}(0),V^{c}(0)\}\in \mathbb{R}^{6}_{+}$, *the solution of model* (3) *has the property*

$$ \begin{aligned} &\limsup_{t\rightarrow \infty }\frac{1}{t}E \int _{0}^{t} \biggl[ \biggl(S^{r}(s)- \frac{\Lambda ^{r}}{\mu +\theta ^{r}} \biggr)^{2}+ \biggl(S^{c}(s)- \frac{\Lambda ^{c}}{\mu +\theta ^{c}} \biggr)^{2}+\bigl(I^{r} \bigr)^{2}(s)+\bigl(I^{c}\bigr)^{2}(s) \\ &\quad\quad {} + \biggl(V^{r}(s)- \frac{\theta ^{r}\Lambda ^{r}}{\mu ^{2}+\mu \theta ^{r}} \biggr)^{2}+ \biggl(V^{c}(s)- \frac{\theta ^{c}\Lambda ^{c}}{\mu ^{2}+\mu \theta ^{c}} \biggr)^{2} \biggr]\,ds \\ &\quad \leq \frac{H}{K^{\prime }}, \end{aligned} $$

*where*

$$ \begin{aligned} K^{\prime }={}&\min \biggl\{ \frac{p\mu (\mu +\theta ^{r})}{\mu +2\theta ^{r}}- \bigl(2\mu +\nu ^{r}\bigr)-(1+P) \sigma _{1}^{2}, \frac{q\mu (\mu +\theta ^{c})}{\mu +2\theta ^{c}}-\bigl(2 \mu +\nu ^{c}\bigr)-(1+q)\sigma _{2}^{2}, \\ &\frac{1}{2}\bigl(\mu +\nu ^{r}-\sigma _{3}\bigr), \frac{1}{2}\bigl(\mu +\nu ^{c}- \sigma _{4}\bigr), \frac{p\mu ^{3}}{2\theta ^{r}(\theta ^{r}+\mu )}-\bigl(2\mu + \nu ^{r}\bigr)- \biggl( \frac{p\mu }{\theta ^{r}}+1 \biggr)\sigma _{5}^{2}, \\ &\frac{q\mu ^{3}}{2\theta ^{c}(\theta ^{c}+\mu )}-\bigl(2\mu +\nu ^{c}\bigr)- \biggl( \frac{p\mu }{\theta ^{c}}+1 \biggr)\sigma _{6}^{2} \biggr\} , \end{aligned} $$

$H=(1+p)\sigma _{1}^{2}a^{2}+(1+q)\sigma _{2}^{2}b^{2}+ ( \frac{p\mu }{\theta ^{r}}+1 )\sigma _{5}^{2}a^{2} \frac{(\theta ^{r})^{2}}{\mu ^{2}}+ (\frac{q\mu }{\theta ^{c}}+1 )\sigma _{6}^{2}b^{2}\frac{(\theta ^{c})^{2}}{\mu ^{2}}$ *and* *p*, *q* *are positive constants defined as* (13), (14).

### Proof

Let $x^{r}=S^{r}-\frac{\Lambda ^{r}}{\mu +\theta ^{r}}$, $x^{c}=S^{c}- \frac{\Lambda ^{c}}{\mu +\theta ^{c}}$, $y^{r}=I^{r}$, $y^{c}=I^{c}$, $z^{r}=V^{r}- \frac{\theta ^{r}\Lambda ^{r}}{\mu ^{2}+\mu \theta ^{r}}$, $z^{c}=V^{c}- \frac{\theta ^{c}\Lambda ^{c}}{\mu ^{2}+\mu \theta ^{c}}$, $a= \frac{\Lambda ^{r}}{\mu +\theta ^{r}}$, $b= \frac{\Lambda ^{c}}{\mu +\theta ^{c}}$, then

$$ \textstyle\begin{cases} dx^{r}(t)= [- (\beta ^{r}_{r}\frac{y^{r}}{1+\alpha _{r}y^{r}}+ \beta ^{r}_{c}\frac{y^{c}}{1+\alpha _{c}y^{c}} )(x^{r}+a)-(\mu + \theta ^{r})x^{r} ]\,dt+\sigma _{1}(a+x^{r})\,dB_{1}(t), \\ dx^{c}(t)= [- (\beta ^{c}_{r}\frac{y^{r}}{1+\alpha _{r}y^{r}}+ \beta ^{c}_{c}\frac{y^{c}}{1+\alpha _{c}y^{c}} )(x^{c}+b)-(\mu + \theta ^{c})x^{c} ]\,dt+\sigma _{2}(b+x^{c})\,dB_{2}(t), \\ dy^{r}(t)= [ (\beta ^{r}_{r}\frac{y^{r}}{1+\alpha _{r}y^{r}}+ \beta ^{r}_{c}\frac{y^{c}}{1+\alpha _{c}y^{c}} )(x^{r}+a)+ ( \kappa ^{r}_{r}\frac{y^{r}}{1+\alpha _{r}y^{r}}+\kappa ^{r}_{c} \frac{y^{c}}{1+\alpha _{c}y^{c}} ) (z^{r}+a \frac{\theta ^{r}}{\mu } ) \\ \hphantom{dy^{r}(t)}\quad{} -(\mu +\nu ^{{r}})y^{r} ]\,dt+\sigma _{3}y^{r}\,dB_{3}(t), \\ dy^{c}(t)= [ (\beta ^{c}_{r}\frac{y^{r}}{1+\alpha _{r}y^{r}}+ \beta ^{c}_{c}\frac{y^{c}}{1+\alpha _{c}y^{c}} )(x^{c}+b)+ ( \kappa ^{c}_{r}\frac{y^{r}}{1+\alpha _{r}y^{r}}+\kappa ^{c}_{c} \frac{y^{c}}{1+\alpha _{c}y^{c}} ) (z^{c}+b \frac{\theta ^{c}}{\mu } ) \\ \hphantom{dy^{c}(t)}\quad{} -(\mu +\nu ^{{c}})y^{c} ]\,dt+\sigma _{4}y^{c}\,dB_{4}(t), \\ dz^{r}(t)= [\theta ^{r}(x^{r}+a)- (\kappa ^{r}_{r} \frac{y^{r}}{1+\alpha _{r}y^{r}}+\kappa ^{r}_{c} \frac{y^{c}}{1+\alpha _{c}y^{c}} ) (z^{r}+a \frac{\theta ^{r}}{\mu } )-\mu (z^{r}+a\frac{\theta ^{r}}{\mu } ) ]\,dt \\ \hphantom{dz^{r}(t)}\quad{} +\sigma _{5} (z^{r}+a\frac{\theta ^{r}}{\mu } )\,dB_{5}(t), \\ dz^{c}(t)= [\theta ^{c}(x^{c}+b)- (\kappa ^{c}_{r} \frac{y^{r}}{1+\alpha _{r}y^{r}}+\kappa ^{c}_{c} \frac{y^{c}}{1+\alpha _{c}y^{c}} ) (z^{c}+b \frac{\theta ^{c}}{\mu } )-\mu (z^{c}+b\frac{\theta ^{c}}{\mu } ) ]\,dt \\ \hphantom{dz^{c}(t)}\quad{} +\sigma _{6} (z^{c}+b\frac{\theta ^{c}}{\mu } )\,dB_{6}(t). \end{cases} $$

Define a $C^{2}$-function *W*: $\mathbb{R}^{6}_{+}\rightarrow \mathbb{R}_{+}$ by

$$ \begin{aligned} W\bigl(x^{r},x^{c},y^{r},y^{c},z^{r},z^{c} \bigr)&= p \biggl(\frac{1}{2}\bigl(x^{r}\bigr)^{2}+c_{1}y^{r}+ \frac{1}{2}c_{2}\bigl(z^{r}\bigr)^{2} \biggr)+\frac{1}{2}\bigl(x^{r},y^{r},z^{r} \bigr)^{2} \\ & \quad {} +q \biggl(\frac{1}{2}\bigl(x^{c}\bigr)^{2}+c_{3}y^{c}+ \frac{1}{2}c_{4}\bigl(z^{c}\bigr)^{2} \biggr)+\frac{1}{2}\bigl(x^{c},y^{c},z^{c} \bigr)^{2} \\ &:=pW_{1}+W_{2}+qW_{3}+W_{4}, \end{aligned} $$

where

$$ \begin{aligned} &W_{1}\bigl(x^{r},x^{c},y^{r},y^{c},z^{r},z^{c} \bigr)= \biggl(\frac{1}{2}\bigl(x^{r}\bigr)^{2}+c_{1}y^{r}+ \frac{1}{2}c_{2}\bigl(z^{r}\bigr)^{2} \biggr), \\ &W_{2}\bigl(x^{r},x^{c},y^{r},y^{c},z^{r},z^{c} \bigr)=\frac{1}{2}\bigl(x^{r},y^{r},z^{r} \bigr)^{2}, \\ &W_{3}\bigl(x^{r},x^{c},y^{r},y^{c},z^{r},z^{c} \bigr)= \biggl(\frac{1}{2}\bigl(x^{c}\bigr)^{2}+c_{3}y^{c}+ \frac{1}{2}c_{4}\bigl(z^{c}\bigr)^{2} \biggr), \\ &W_{4}\bigl(x^{r},x^{c},y^{r},y^{c},z^{r},z^{c} \bigr)=\frac{1}{2}\bigl(x^{c},y^{c},z^{c} \bigr)^{2}, \end{aligned} $$

and *p*, *q* and $c_{1}$, $c_{2}$, $c_{3}$, $c_{4}$ are positive constants. From *Itô*’s formula, we compute

$$ \begin{aligned} dW_{1}\bigl(x^{r},y^{r},z^{r} \bigr)={}&LW_{1}\,dt+\sigma _{1}x^{r} \bigl(x^{r}+a\bigr)\,dB_{1}(t)+c_{1} \sigma _{3}y^{r}\,dB_{3}(t) \\ &{}+c_{2}z^{r} \sigma _{5} \biggl(z^{r}+a \frac{\theta ^{r}}{\mu } \biggr) \,dB_{5}(t), \end{aligned} $$

where

$$\begin{aligned} LW_{1}={}&-x^{r} \biggl(\beta ^{r}_{r}\frac{y^{r}}{1+\alpha _{r}y^{r}}+ \beta ^{r}_{c} \frac{y^{c}}{1+\alpha _{c}y^{c}} \biggr) \bigl(x^{r}+a\bigr)-\bigl(\mu + \theta ^{r}\bigr) \bigl(x^{r}\bigr)^{2} \\ & {} +\frac{1}{2}\sigma ^{2}_{1} \bigl(a+x^{r}\bigr)^{2}\,dB_{1}(t)+c_{1} \biggl(\kappa ^{r}_{r} \frac{y^{r}}{1+\alpha _{r}y^{r}}+\kappa ^{r}_{c} \frac{y^{c}}{1+\alpha _{c}y^{c}} \biggr) \biggl(z^{r}+a \frac{\theta ^{r}}{\mu } \biggr) \\ & {} +c_{1} \biggl(\beta ^{r}_{r} \frac{y^{r}}{1+\alpha _{r}y^{r}}+\beta ^{r}_{c} \frac{y^{c}}{1+\alpha _{c}y^{c}} \biggr) \bigl(x^{r}+a\bigr)-c_{1}\bigl(\mu +\nu ^{r} \bigr)y^{r}+c_{2}z^{r} \theta ^{r} \bigl(x^{r}+a\bigr) \\ & {} -c_{2}z^{r} \biggl(\kappa ^{r}_{r} \frac{y^{r}}{1+\alpha _{r}y^{r}}+ \kappa ^{r}_{c}\frac{y^{c}}{1+\alpha _{c}y^{c}} \biggr) \biggl(z^{r}+a \frac{\theta ^{r}}{\mu } \biggr)-c_{2}z^{r} \mu \biggl(z^{r}+a \frac{\theta ^{r}}{\mu } \biggr) \\ & {} +\frac{1}{2}c_{2}\sigma _{5}^{2} \biggl(z^{r}+a\frac{\theta ^{r}}{\mu } \biggr)^{2} \,dB_{5}(t). \end{aligned}$$

For the convenience of calculation, we simplify $\frac{x}{1+\alpha _{i}x}$ ($i=r,c$) to *x*, then

$$ \begin{aligned} LW_{1}={}&-x^{r}\bigl(\beta ^{r}_{r}y^{r}+\beta ^{r}_{c}y^{c} \bigr) \bigl(x^{r}+a\bigr)-\bigl(x^{r}\bigr)^{2} \bigl( \mu +\theta ^{r}\bigr)+\frac{1}{2}\sigma ^{2}_{1}\bigl(a+x^{r}\bigr)^{2} \,dB_{1}(t) \\ & {} +c_{1}\bigl(\beta ^{r}_{r}y^{r}+ \beta ^{r}_{c}y^{c}\bigr) \bigl(x^{r}+a \bigr)+c_{1}\bigl( \kappa ^{r}_{r}y^{r}+ \kappa ^{r}_{c}y^{c}\bigr) \biggl(z^{r}+a \frac{\theta ^{r}}{\mu } \biggr)-c_{1}\bigl(\mu +\nu ^{r} \bigr)y^{r} \\ & {} +c_{2}z^{r}\theta ^{r}\bigl(x^{r}+a \bigr)-c_{2}z^{r}\bigl(\kappa ^{r}_{r}y^{r}+ \kappa ^{r}_{c}y^{c}\bigr) \biggl(z^{r}+a \frac{\theta ^{r}}{\mu } \biggr)-c_{2}z^{r} \mu \biggl(z^{r}+a\frac{\theta ^{r}}{\mu } \biggr) \\ & {} +\frac{1}{2}c_{2}\sigma _{5}^{2} \biggl(z^{r}+a\frac{\theta ^{r}}{\mu } \biggr)^{2} \,dB_{5}(t). \end{aligned} $$

Assume

$$ \begin{aligned} &R_{0}^{r}=\beta ^{r}_{r} \frac{\Lambda ^{r}}{(\mu +\theta ^{r})(\mu +\nu ^{r})}+\kappa ^{r}_{r} \frac{1}{\mu +\nu ^{r}} \frac{\theta ^{r}\Lambda ^{r}}{\mu ^{2}+\mu \theta ^{r}}, \end{aligned} $$

so

$$ \begin{aligned} &R_{0}^{r}\leq \rho \bigl(FV^{-1}\bigr)=R_{0}\leq 1. \end{aligned} $$

Then

9 $$\begin{aligned} \begin{aligned} LW_{1}={}&-\beta ^{r}_{r} \bigl(x^{r}\bigr)^{2} y^{r}-\beta ^{r}_{c}\bigl(x^{r}\bigr)^{2} y^{c}-c_{2} \kappa ^{r}_{r} y^{r}\bigl(z^{r}\bigr)^{2}-c_{2}\kappa ^{r}_{c} y^{c}\bigl(z^{r} \bigr)^{2}-\bigl( \mu +\theta ^{r}\bigr) \bigl(x^{r}\bigr)^{2} \\ & {} +(c_{1}-a)\beta ^{r}_{r}x^{r}y^{r}+(c_{1}-a) \beta ^{r}_{c}x^{r}y^{c}+ \biggl(c_{1}-c_{2}\frac{a\theta ^{r}}{\mu } \biggr)\kappa ^{r}_{r}y^{r}z^{r}+ \biggl(c_{1}-c_{2} \frac{a\theta ^{r}}{\mu } \biggr)\kappa ^{r}_{c}y^{c}z^{r} \\ & {} +c_{2}\theta ^{r}x^{r}z^{r}-c_{2} \mu \bigl(z^{r}\bigr)^{2}-c_{1} \biggl[\beta ^{r}_{r}a \biggl(\frac{1}{R_{0}^{r}}-1 \biggr)+ \frac{a\theta ^{r}\kappa ^{r}_{r}}{\mu } \biggl(\frac{1}{R_{0}^{r}}-1 \biggr) \biggr]y^{r} \\ & {} +\frac{1}{2}\sigma _{1}^{2} \bigl(a+x^{r}\bigr)^{2}\,dB_{1}(t)+ \frac{1}{2}c_{2} \sigma _{5}^{2} \biggl(z^{r}+a\frac{\theta ^{r}}{\mu } \biggr)^{2} \,dB_{5}(t), \end{aligned} \end{aligned}$$

where $R_{0}\leq 1$ is used. Here we choose $c_{1}=a$, $c_{2}= \frac{\mu }{\theta ^{r}}$, and let $h=\frac{\mu (\mu +2\theta ^{r})}{2\theta ^{r}(\mu +\theta ^{r})}$, substituting this into (9) yields

$$ \begin{aligned} LW_{1}\leq{}&- \biggl[\bigl(\mu +\theta ^{r}\bigr)-\frac{\mu }{h} \biggr]\bigl(x^{r} \bigr)^{2}- \biggl[\frac{\mu ^{2}}{\theta ^{r}}-h\mu \biggr]\bigl(z^{r} \bigr)^{2}+\sigma ^{2}_{1}\bigl(x^{r} \bigr)^{2}+ \frac{\mu }{\theta ^{r}}\sigma ^{2}_{5} \bigl(z^{r}\bigr)^{2} \\ & {} +\sigma ^{2}_{1}a^{2}+\frac{\mu }{\theta ^{r}} \sigma _{5}^{2}a^{2} \frac{(\theta ^{r})^{2}}{\mu ^{2}} \\ ={}&-\frac{\mu (\mu +\theta ^{r})}{\mu +2\theta ^{r}}\bigl(x^{r}\bigr)^{2}- \frac{\mu ^{3}}{2\theta ^{r}(\mu +\theta ^{r})}\bigl(z^{r}\bigr)^{2}+\sigma ^{2}_{1}\bigl(x^{r}\bigr)^{2}+ \frac{\mu }{\theta ^{r}}\sigma ^{2}_{5}\bigl(z^{r} \bigr)^{2} \\ & {} +\sigma ^{2}_{1}a^{2}+\frac{\mu }{\theta ^{r}} \sigma _{5}^{2}a^{2} \frac{(\theta ^{r})^{2}}{\mu ^{2}}. \end{aligned} $$

Similarly, applying *Itô*’s formula to $W_{2}(x^{r},y^{r},z^{r})$, we get

$$ \begin{aligned}& dW_{2}\bigl(x^{r},y^{r},z^{r} \bigr)\\&\quad =LW_{2}\,dt+\bigl(x^{r},y^{r},z^{r} \bigr) \biggl[\sigma _{1}\bigl(a+x^{r}\bigr) \,dB_{1}(t)+ \sigma _{3}y^{r}\,dB_{3}(t)+ \sigma _{5} \biggl(z^{r}+a \frac{\theta ^{r}}{\mu } \biggr) \,dB_{5}(t) \biggr], \end{aligned} $$

where

$$ \begin{aligned} LW_{2}&=\bigl(x^{r},y^{r},z^{r} \bigr) \bigl(dx^{r}+dy^{r}+dz^{r}\bigr)+ \frac{1}{2}\bigl[\bigl(dx^{r}\bigr)^{2}+ \bigl(dy^{r}\bigr)^{2}+\bigl(dz^{r} \bigr)^{2}\bigr] \\ &=\bigl(x^{r},y^{r},z^{r}\bigr) \biggl[-x^{r}\bigl(\mu +\theta ^{r}\bigr)-y^{r} \bigl(\mu +\nu ^{r}\bigr)- \mu \biggl(z^{r}+a \frac{\theta ^{r}}{\mu } \biggr)+\theta ^{r}\bigl(x^{r}+a\bigr) \biggr] \\ & \quad {} +\frac{1}{2}\sigma ^{2}_{1} \bigl(x^{r}+a\bigr)^{2}+\frac{1}{2}\sigma ^{2}_{3}\bigl(y^{r}\bigr)^{2}+ \frac{1}{2}\sigma ^{2}_{5} \biggl(z^{r}+a \frac{\theta ^{r}}{\mu } \biggr)^{2} \\ &\leq \bigl(2\mu +\nu ^{r}\bigr) \bigl(x^{r} \bigr)^{2}-\frac{\mu +\nu ^{r}}{2}\bigl(y^{r}\bigr)^{2}+ \bigl(2 \mu +\nu ^{r}\bigr) \bigl(z^{r}\bigr)^{2}+ \sigma ^{2}_{1}\bigl(x^{r}\bigr)^{2}+ \frac{1}{2} \sigma ^{2}_{3}\bigl(y^{r} \bigr)^{2} \\ &\quad {} +\sigma ^{2}_{5}\bigl(z^{r} \bigr)^{2}+\sigma ^{2}_{1}a^{2}+\sigma ^{2}_{5}a^{2} \frac{(\theta ^{r})^{2}}{\mu ^{2}}. \end{aligned} $$

Then

$$ \begin{aligned} dW_{3}\bigl(x^{c},y^{c},z^{c} \bigr)={}&LW_{3}\,dt+\sigma _{2}x^{c} \bigl(x^{c}+b\bigr)\,dB_{2}(t)+c_{3} \sigma _{4}y^{c}\,dB_{4}(t) \\ &{}+c_{4}z^{c} \sigma _{6} \biggl(z^{c}+b \frac{\theta ^{c}}{\mu } \biggr) \,dB_{6}(t). \end{aligned} $$

Assume

$$ \begin{aligned} &R_{0}^{c}=\beta ^{c}_{c} \frac{\Lambda ^{c}}{(\mu +\theta ^{c})(\mu +\nu ^{c})}+\kappa ^{c}_{c} \frac{1}{\mu +\nu ^{c}} \frac{\theta ^{c}\Lambda ^{c}}{\mu ^{2}+\mu \theta ^{c}}, \end{aligned} $$

so

$$ \begin{aligned} &R_{0}^{c}\leq \rho \bigl(FV^{-1}\bigr)=R_{0}\leq 1, \end{aligned} $$

then

10 $$\begin{aligned} \begin{aligned} LW_{3}={}&-\beta ^{c}_{r} \bigl(x^{c}\bigr)^{2} y^{r}-\beta ^{c}_{c}\bigl(x^{c}\bigr)^{2} y^{c}-c_{4} \kappa ^{c}_{r} y^{r}\bigl(z^{c}\bigr)^{2}-c_{4}\kappa ^{c}_{c} y^{c}\bigl(z^{c} \bigr)^{2}-\bigl( \mu +\theta ^{c}\bigr) \bigl(x^{c}\bigr)^{2} \\ & {} +(c_{3}-b)\beta ^{c}_{r}x^{c}y^{r}+(c_{3}-b) \beta ^{c}_{c}x^{c}y^{c}+ \biggl(c_{3}-c_{4}\frac{b\theta ^{c}}{\mu } \biggr)\kappa ^{c}_{r}y^{r}z^{c}+ \biggl(c_{3}-c_{4} \frac{b\theta ^{c}}{\mu } \biggr)\kappa ^{c}_{c}y^{c}z^{c} \\ & {} +c_{4}\theta ^{c}x^{c}z^{c}-c_{4} \mu \bigl(z^{c}\bigr)^{2}-c_{3} \biggl[\beta ^{c}_{c}b \biggl(\frac{1}{R_{0}^{c}}-1 \biggr)+ \frac{b\theta ^{c}\kappa ^{c}_{c}}{\mu } \biggl(\frac{1}{R_{0}^{c}}-1 \biggr) \biggr]y^{c} \\ & {} +\frac{1}{2}\sigma _{2}^{2} \bigl(b+x^{c}\bigr)^{2} \,dB_{2}(t)+ \frac{1}{2}c_{4} \sigma _{6}^{2} \biggl(z^{c}+b\frac{\theta ^{c}}{\mu } \biggr)^{2} \,dB_{6}(t), \end{aligned} \end{aligned}$$

where $R_{0}\leq 1$ is used. Here we choose $c_{2}=b$, $c_{4}= \frac{\mu }{\theta ^{c}}$, and let $h'=\frac{\mu (\mu +2\theta ^{c})}{2\theta ^{c}(\mu +\theta ^{c})}$, substituting this into (10) yields

$$\begin{aligned} LW_{3}\leq{}&- \biggl[\bigl(\mu +\theta ^{c}\bigr)-\frac{\mu }{h'} \biggr]\bigl(x^{c} \bigr)^{2}- \biggl[\frac{\mu ^{2}}{\theta ^{c}}-h'\mu \biggr] \bigl(z^{c}\bigr)^{2}+\sigma ^{2}_{2} \bigl(x^{c}\bigr)^{2}+ \frac{\mu }{\theta ^{c}}\sigma ^{2}_{6}\bigl(z^{c}\bigr)^{2} \\ & {} +\sigma ^{2}_{2}b^{2}+\frac{\mu }{\theta ^{c}} \sigma _{6}^{2}b^{2} \frac{(\theta ^{c})^{2}}{\mu ^{2}} \\ ={}&-\frac{\mu (\mu +\theta ^{c})}{\mu +2\theta ^{c}}\bigl(x^{c}\bigr)^{2}- \frac{\mu ^{3}}{2\theta ^{c}(\mu +\theta ^{c})}\bigl(z^{c}\bigr)^{2}+\sigma ^{2}_{2}\bigl(x^{c}\bigr)^{2}+ \frac{\mu }{\theta ^{c}}\sigma ^{2}_{6}\bigl(z^{c} \bigr)^{2} \\ & {} +\sigma ^{2}_{2}b^{2}+\frac{\mu }{\theta ^{c}} \sigma _{6}^{2}b^{2} \frac{(\theta ^{c})^{2}}{\mu ^{2}}. \end{aligned}$$

Similarly, applying *Itô*’s formula to $W_{4}(x^{c},y^{c},z^{c})$, we get

$$ \begin{aligned} &dW_{4}\bigl(x^{c},y^{c},z^{c} \bigr) \\ &\quad =LW_{4}\,dt+\bigl(x^{c},y^{c},z^{c} \bigr)\biggl[\sigma _{2}\bigl(b+x^{c}\bigr) \,dB_{2}(t)+\sigma _{4}y^{c}\,dB_{4}(t)+ \sigma _{6} \biggl(z^{c}+b\frac{\theta ^{c}}{\mu } \biggr) \,dB_{6}(t)\biggr], \end{aligned} $$

where

11 $$ \begin{aligned} LW_{4}&=\bigl(x^{c},y^{c},z^{c} \bigr) \bigl(dx^{c}+dy^{c}+dz^{c}\bigr)+ \frac{1}{2}\bigl[\bigl(dx^{c}\bigr)^{2}+ \bigl(dy^{c}\bigr)^{2}+\bigl(dz^{c} \bigr)^{2}\bigr] \\ &=\bigl(x^{c},y^{c},z^{c}\bigr) \biggl[-x^{c}\bigl(\mu +\theta ^{c}\bigr)-y^{c} \bigl(\mu +\nu ^{c}\bigr)- \mu \biggl(z^{c}+b \frac{\theta ^{c}}{\mu } \biggr)+\theta ^{c}\bigl(x^{c}+b\bigr) \biggr] \\ &\quad {} +\frac{1}{2}\sigma ^{2}_{2} \bigl(x^{c}+b\bigr)^{2}+\frac{1}{2}\sigma ^{2}_{4}\bigl(y^{c}\bigr)^{2}+ \frac{1}{2}\sigma ^{2}_{6} \biggl(z^{c}+b \frac{\theta ^{c}}{\mu } \biggr)^{2} \\ &\leq \bigl(2\mu +\nu ^{c}\bigr) \bigl(x^{c} \bigr)^{2}-\frac{\mu +\nu ^{c}}{2}\bigl(y^{c}\bigr)^{2}+ \bigl(2 \mu +\nu ^{c}\bigr) \bigl(z^{c}\bigr)^{2}+ \sigma ^{2}_{2}\bigl(x^{c}\bigr)^{2}+ \frac{1}{2} \sigma ^{2}_{4}\bigl(y^{c} \bigr)^{2} \\ &\quad {} +\sigma ^{2}_{6}\bigl(z^{c} \bigr)^{2}+\sigma ^{2}_{2}b^{2}+\sigma ^{2}_{6}b^{2} \frac{(\theta ^{c})^{2}}{\mu ^{2}}. \end{aligned} $$

Therefore, we can obtain

12 $$ \begin{aligned} &LW\bigl(x^{r},x^{c},y^{r},y^{c},z^{r},z^{c} \bigr) \\ &\quad =pW_{1}+W_{2}+qW_{3}+W_{4} \\ &\quad \quad{} - \biggl[p\frac{\mu (\mu +\theta ^{r})}{\mu +2\theta ^{r}}-\bigl(2\mu +\nu ^{r}\bigr)-(1+p) \sigma _{1}^{2} \biggr]\bigl(x^{r} \bigr)^{2}- \biggl[q \frac{\mu (\mu +\theta ^{c})}{\mu +2\theta ^{c}}-\bigl(2\mu +\nu ^{c} \bigr) \\ &\quad \quad{}-(1+q)\sigma _{2}^{2} \biggr]\bigl(x^{c} \bigr)^{2}-\frac{1}{2}\bigl(\mu +\nu ^{r}- \sigma _{3}\bigr) \bigl(y^{r}\bigr)^{2}- \frac{1}{2}\bigl(\mu +\nu ^{c}-\sigma _{4}\bigr) \bigl(y^{c}\bigr)^{2} \\ &\quad \quad{}- \biggl[p\frac{\mu ^{3}}{2\theta ^{r}(\theta ^{r}+\mu )}-\bigl(2\mu +\nu ^{r}\bigr)- \biggl( \frac{p\mu }{\theta ^{r}}+1\biggr)\sigma _{5}^{2} \biggr] \bigl(z^{r}\bigr)^{2} \\ &\quad \quad{}- \biggl[q\frac{\mu ^{3}}{2\theta ^{c}(\theta ^{c}+\mu )}-\bigl(2\mu +\nu ^{c}\bigr)- \biggl( \frac{q\mu }{\theta ^{c}}+1\biggr)\sigma _{6}^{2} \biggr] \bigl(z^{c}\bigr)^{2}+H, \end{aligned} $$

where $H=(1+p)\sigma _{1}^{2}a^{2}+(1+q)\sigma _{2}^{2}b^{2}+( \frac{p\mu }{\theta ^{r}}+1)\sigma _{5}^{2}a^{2} \frac{(\theta ^{r})^{2}}{\mu ^{2}}+(\frac{q\mu }{\theta ^{c}}+1) \sigma _{6}^{2}b^{2}\frac{(\theta ^{c})^{2}}{\mu ^{2}}$. Let us choose

13 $$ p>\max \biggl\{ \frac{\mu (\mu +\theta ^{r})}{\mu +2\theta ^{r}}\bigl(2\mu + \nu ^{r} \bigr),\frac{\mu ^{3}}{2\theta ^{r}(\theta ^{r}+\mu )}\bigl(2\mu +\nu ^{r}\bigr) \biggr\} , $$

s.t.

14 $$\begin{aligned}& p\frac{\mu (\mu +\theta ^{r})}{\mu +2\theta ^{r}}-\bigl(2\mu +\nu ^{r}\bigr)>0, \quad \quad p\frac{\mu ^{3}}{2\theta ^{r}(\theta ^{r}+\mu )}-\bigl(2\mu +\nu ^{r}\bigr)>0. \\& q>\max \biggl\{ \frac{\mu (\mu +\theta ^{c})}{\mu +2\theta ^{c}}\bigl(2\mu + \nu ^{c}\bigr), \frac{\mu ^{3}}{2\theta ^{c}(\theta ^{c}+\mu )}\bigl(2\mu +\nu ^{c}\bigr) \biggr\} , \end{aligned}$$

s.t.

$$ \begin{aligned} &q\frac{\mu (\mu +\theta ^{c})}{\mu +2\theta ^{c}}-\bigl(2\mu +\nu ^{c} \bigr)>0, \quad\quad q \frac{\mu ^{3}}{2\theta ^{c}(\theta ^{c}+\mu )}-\bigl(2\mu +\nu ^{c}\bigr)>0. \end{aligned} $$

Set

$$ \begin{aligned} K'={}&\min \biggl\{ \frac{p\mu (\mu +\theta ^{r})}{\mu +2\theta ^{r}}- \bigl(2 \mu +\nu ^{r}\bigr)-(1+p)\sigma _{1}^{2}, \frac{q\mu (\mu +\theta ^{c})}{\mu +2\theta ^{c}}-\bigl(2\mu +\nu ^{c}\bigr)-(1+q) \sigma _{2}^{2}, \\ &\frac{1}{2}\bigl(\mu +\nu ^{r}-\sigma _{3}\bigr), \frac{1}{2}\bigl(\mu +\nu ^{c}- \sigma _{4}\bigr), \frac{p\mu ^{3}}{2\theta ^{r}(\theta ^{r}+\mu )}-\bigl(2\mu + \nu ^{r}\bigr)-\biggl( \frac{p\mu }{\theta ^{r}}+1\biggr)\sigma _{5}^{2}, \\ &\frac{q\mu ^{3}}{2\theta ^{c}(\theta ^{c}+\mu )}-\bigl(2\mu +\nu ^{c}\bigr)-\biggl( \frac{q\mu }{\theta ^{c}}+1\biggr)\sigma _{6}^{2} \biggr\} . \end{aligned} $$

Substituting this into (12) yields

15 $$ \begin{aligned} dW\leq{}& \bigl[-K'\bigl(x^{r} \bigr)^{2}-K'\bigl(x^{c}\bigr)^{2}-K' \bigl(y^{r}\bigr)^{2}-K'\bigl(y^{c} \bigr)^{2}-K'\bigl(z^{r}\bigr)^{2}-K' \bigl(z^{c}\bigr)^{2}+H\bigr]\,dt \\ & {} +\sigma _{1}\bigl[(p+1)x^{r}+y^{r}+z^{r} \bigr]\bigl(a+x^{r}\bigr)\,dB_{1}(t)+\bigl[x^{r}+y^{r}+z^{r}+c_{1}p \bigr] \sigma _{3}y^{r}\,dB_{3}(t) \\ & {} +\sigma _{2}\bigl[(q+1)x^{c}+y^{c}+z^{c} \bigr]\bigl(b+x^{c}\bigr)\,dB_{2}(t)+\bigl[x^{c}+y^{c}+z^{c}+c_{3}q \bigr] \sigma _{4}y^{c}\,dB_{4}(t) \\ & {} +\bigl[x^{r}+y^{r}+z^{r}+c_{2}p z^{r}\bigr]\sigma _{5} \biggl(z^{r}+a \frac{\theta ^{r}}{\mu } \biggr)\,dB_{5}(t) \\ & {} +\bigl[x^{c}+y^{c}+z^{c}+c_{4}q z^{c}\bigr]\sigma _{6} \biggl(z^{c}+b \frac{\theta ^{c}}{\mu } \biggr)\,dB_{6}(t). \end{aligned} $$

Integrating this from 0 to *t* and taking the expectation, we have

$$ \begin{aligned} EW(t)-EW(0)\leq{}&-E \int _{0}^{t}\bigl(K' \bigl(x^{r}\bigr)^{2}+K'\bigl(x^{c} \bigr)^{2}+K'\bigl(y^{r}\bigr)^{2}+K' \bigl(y^{c}\bigr)^{2}+K'\bigl(z^{r} \bigr)^{2} \\ & {} +K'\bigl(z^{c}\bigr)^{2}+H\bigr)\,ds. \end{aligned} $$

Hence

$$ \begin{aligned} &\limsup_{t\rightarrow \infty }\frac{1}{t}E \int _{0}^{t}\bigl[\bigl(x^{r} \bigr)^{2}(s)+\bigl(x^{c}\bigr)^{2}(s)+ \bigl(y^{r}\bigr)^{2}(s)+\bigl(y^{c} \bigr)^{2}(s)+\bigl(z^{r}\bigr)^{2}(s)+ \bigl(z^{c}\bigr)^{2}(s)\bigr]\,ds \leq \frac{H}{K'}. \end{aligned} $$

Consequently,

$$ \begin{aligned} &\limsup_{t\rightarrow \infty }\frac{1}{t}E \int _{0}^{t} \biggl[ \biggl(S^{r}(s)- \frac{\Lambda ^{r}}{\mu +\theta ^{r}} \biggr)^{2}+ \biggl(S^{c}(s)- \frac{\Lambda ^{c}}{\mu +\theta ^{c}} \biggr)^{2}+\bigl(I^{r} \bigr)^{2}(s)+\bigl(I^{c}\bigr)^{2}(s) \\ &\quad\quad{} + \biggl(V^{r}(s)- \frac{\theta ^{r}\Lambda ^{r}}{\mu ^{2}+\mu \theta ^{r}} \biggr)^{2}+ \biggl(V^{c}(s)- \frac{\theta ^{c}\Lambda ^{c}}{\mu ^{2}+\mu \theta ^{c}} \biggr)^{2} \biggr]\,ds \\ &\quad \leq \frac{H}{K^{\prime }}. \end{aligned} $$

 □

### Remark 5.1

From Theorem 5.1, we show that the solution of system (3) will oscillate around the disease-free equilibrium of the deterministic model under some conditions, and the disturbance intensity is proportional to the intensity of the white noise. In a biological view, as the intensity of stochastic perturbations is small, we consider the disease will die out.

Besides, if $\sigma _{1}=0$, $\sigma _{2}=0$, $\sigma _{5}=0$, and $\sigma _{6}=0$, then $E_{0}$ is also the disease-free equilibrium of system (3). From the proof of Theorem 5.1, we get

$$ \begin{aligned} &LW\bigl(x^{r},x^{c},y^{r},y^{c},z^{r},z^{c} \bigr) \\ &\quad \leq - \biggl[p \frac{\mu (\mu +\theta ^{r})}{\mu +2\theta ^{r}}-\bigl(2\mu +\nu ^{r} \bigr)-(1+p) \sigma _{1}^{2} \biggr]\bigl(x^{r} \bigr)^{2} \\ &\quad \quad{} - \biggl[q\frac{\mu (\mu +\theta ^{c})}{\mu +2\theta ^{c}}-\bigl(2\mu +\nu ^{c}\bigr)-(1+q) \sigma _{2}^{2} \biggr]\bigl(x^{c} \bigr)^{2}-\frac{1}{2}\bigl(\mu +\nu ^{r}-\sigma _{3}\bigr) \bigl(y^{r}\bigr)^{2} \\ &\quad \quad{}- \biggl[p\frac{\mu ^{3}}{2\theta ^{r}(\theta ^{r}+\mu )}-\bigl(2\mu +\nu ^{r}\bigr)-v \biggl(\frac{p\mu }{\theta ^{r}}+1 \biggr)\sigma _{5}^{2} \biggr] \bigl(z^{r}\bigr)^{2}- \frac{1}{2}\bigl(\mu +\nu ^{c}-\sigma _{4}\bigr) \bigl(y^{c} \bigr)^{2} \\ &\quad \quad{}- \biggl[q\frac{\mu ^{3}}{2\theta ^{c}(\theta ^{c}+\mu )}-\bigl(2\mu +\nu ^{c}\bigr)- \biggl(\frac{q\mu }{\theta ^{c}}+1 \biggr)\sigma _{6}^{2} \biggr] \bigl(z^{c}\bigr)^{2}. \end{aligned} $$

Thus, the solution of system (3) is stochastically asymptotically stable in the large.

## Numerical simulation

In this subsection, in order to show different dynamical results of the deterministic model (2) and its stochastic description (3) under the same condition of parameter values, we present some numerical simulations. We use Milstein’s method [32, 33] to simulate the stochastic model (3). The numerical scheme for the stochastic model (3) is given by

$$ \textstyle\begin{cases} S^{r}_{i}=S^{r}_{i-1}+ [\Lambda ^{r}- (\beta ^{r}_{r} \frac{(I^{r})_{i-1}}{1+\alpha _{r}(I^{r})_{i-1}}+\beta ^{r}_{c} \frac{(I^{c})_{i-1}}{1+\alpha _{c}(I^{c})_{i-1}} )(S^{r})_{i-1}-( \mu +\theta ^{r})(S^{r})_{i-1} ]\Delta t \\ \hphantom{S^{r}_{i}}\quad{} +\sigma _{1}(S^{r})_{i-1}\sqrt{\Delta t}\xi _{i-1}+ \frac{\sigma _{1}^{2}}{2}(S^{r})_{i-1}(\xi _{i-1}^{2}-1)\Delta t, \\ S^{c}_{i}=S^{c}_{i-1}+ [\Lambda ^{c}- (\beta ^{c}_{r} \frac{(I^{r})_{i-1}}{1+\alpha _{r}(I^{r})_{i-1}}+\beta ^{c}_{c} \frac{(I^{c})_{i-1}}{1+\alpha _{c}(I^{c})_{i-1}} )(S^{c})_{i-1}-( \mu +\theta ^{c})(S^{c})_{i-1} ]\Delta t \\ \hphantom{S^{c}_{i}}\quad{} +\sigma _{2}(S^{c})_{i-1}\sqrt{\Delta t}\xi _{i-1}+ \frac{\sigma _{2}^{2}}{2}(S^{c})_{i-1}(\xi _{i-1}^{2}-1)\Delta t, \\ I^{r}_{i}=I^{r}_{i-1}+ [ (\beta ^{r}_{r} \frac{(I^{r})_{i-1}}{1+\alpha _{r}(I^{r})_{i-1}}+\beta ^{r}_{c} \frac{(I^{c})_{i-1}}{1+\alpha _{c}(I^{c})_{i-1}} )(S^{r})_{i-1}+ (\kappa ^{r}_{r}\frac{(I^{r})_{i-1}}{1+\alpha _{r}(I^{r})_{i-1}}+ \kappa ^{r}_{c}\frac{(I^{c})_{i-1}}{1+\alpha _{c}(I^{c})_{i-1}} ) \\ \hphantom{I^{r}_{i}}\quad{}\times (V^{r})_{i-1}-(\mu +\nu ^{{r}})(I^{r})_{i-1} ]\Delta t+ \sigma _{3}(I^{c})_{i-1}\sqrt{\Delta t}\xi _{i-1}+ \frac{\sigma _{3}^{2}}{2}(I^{r})_{i-1}(\xi _{i-1}^{2}-1)\Delta t, \\ I^{c}_{i}=I^{c}_{i-1}+ [ (\beta ^{c}_{r} \frac{(I^{r})_{i-1}}{1+\alpha _{r}(I^{r})_{i-1}}+\beta ^{c}_{c} \frac{(I^{c})_{i-1}}{1+\alpha _{c}(I^{c})_{i-1}} )(S^{c})_{i-1}+ (\kappa ^{c}_{r}\frac{(I^{r})_{i-1}}{1+\alpha _{r}(I^{r})_{i-1}}+ \kappa ^{c}_{c}\frac{(I^{c})_{i-1}}{1+\alpha _{c}(I^{c})_{i-1}} ) \\ \hphantom{I^{c}_{i}}\quad{}\times (V^{c})_{i-1}-(\mu +\nu ^{{c}})(I^{c})_{i-1} ]\Delta t+ \sigma _{4}(I^{c})_{i-1}\sqrt{\Delta t}\xi _{i-1}+ \frac{\sigma _{4}^{2}}{2}(I^{c})_{i-1}(\xi _{i-1}^{2}-1)\Delta t, \\ V^{r}_{i}=V^{r}_{i-1}+ [\theta ^{r}(S^{r})_{i-1}- (\kappa ^{r}_{r} \frac{(I^{r})_{i-1}}{1+\alpha _{r}(I^{r})_{i-1}}+\kappa ^{r}_{c} \frac{(I^{c})_{i-1}}{1+\alpha _{c}(I^{c})_{i-1}} )(V^{r})_{i-1}- \mu (V^{r})_{i-1} ]\Delta t \\ \hphantom{V^{r}_{i}}\quad{} +\sigma _{5}(V^{r})_{i-1}\sqrt{\Delta t}\xi _{i-1}+ \frac{\sigma _{5}^{2}}{2}(V^{r})_{i-1}(\xi _{i-1}^{2}-1)\Delta t, \\ V^{c}_{i}=V^{c}_{i-1}+ [\theta ^{c}(S^{c})_{i-1}- (\kappa ^{c}_{r} \frac{(I^{r})_{i-1}}{1+\alpha _{r}(I^{r})_{i-1}}+\kappa ^{c}_{c} \frac{(I^{c})_{i-1}}{1+\alpha _{c}(I^{c})_{i-1}} )(V^{c})_{i-1}- \mu (V^{c})_{i-1} ]\Delta t \\ \hphantom{V^{c}_{i}}\quad{} +\sigma _{6}(V^{c})_{i-1}\sqrt{\Delta t}\xi _{i-1}+ \frac{\sigma _{6}^{2}}{2}(V^{c})_{i-1}(\xi _{i-1}^{2}-1)\Delta t, \end{cases} $$

where $\xi _{i}$ ($i=1,2,\ldots,n$) are independent Gaussian random variables $N(0,1)$.

For the deterministic model (2) and its stochastic description (3), the parameter values are taken as in Table 1.

**Table 1:** Parameter values of numerical experiments for model (2) and model (3)

Parameter	Meaning	Value
$\Lambda ^{r}$	Population input for group *r*	1.005
$\Lambda ^{c}$	Population input for group *c*	5
*μ*	Natural death rate	1/70
$\nu ^{{r}}$	Disease-induced death rate in group *r*	0.4
$\nu ^{{c}}$	Disease-induced death rate in group *c*	0.1
$\beta ^{r}_{r}$	Contact rate of risky susceptible and risky infective	9 × 10^−5^
$\beta ^{r}_{c}$	Contact rate of risky susceptible and critical infective	3 × 10^−5^
$\beta ^{c}_{r}$	Contact rate of critical susceptible and risky infective	3 × 10^−5^
$\beta ^{c}_{c}$	Contact rate of critical susceptible and critical infective	1 × 10^−5^
$\kappa ^{r}_{r}$	Contact rate of risky vaccinated individuals and risky infective	5 × 10^−7^
$\kappa ^{r}_{c}$	Contact rate of risky vaccinated individuals and critical infective	3 × 10^−7^
$\kappa ^{c}_{r}$	Contact rate of critical vaccinated individuals and risky infective	3 × 10^−7^
$\kappa ^{c}_{c}$	Contact rate of critical vaccinated individuals and critical infective	1 × 10^−7^
$\alpha _{i}$	where parameters $\alpha _{i}$ is positive constant (*i* = *r*,*c*)	1 × 10^−5^
$\theta ^{i}$	Vaccination rate (*i* = *r*,*c*)	0.5

The initial value in the numerical experiment is $(S^{r}_{0} , S^{c}_{0} , I^{r}_{0} , I^{c}_{0} , V^{r}_{0} , V^{c}_{0}) = (0.1,0.1,0.1,0.1, 0,0)$. We first choose $p=(99{,}400)>(3.10434\times 10^{-3})=\max \{3.10434\times 10^{-3},2.42954 \times 10^{-6} \}$, $q=(99{,}400)>(9.31301\times 10^{-4})=\max \{9.31301\times 10^{-4},7.28861 \times 10^{-7}\}$, then $R_{0}\approx 0.0017<1$, and $\sigma _{1}=(0.085)$, $\sigma _{2}=(0.085)$, $\sigma _{3}=(0.63)$, $\sigma _{4}=(0.338)$, $\sigma _{5}=(6.84\times 10^{-3})$, $\sigma _{6}=(1.233\times 10^{-2})$. It satisfies the constraint condition of Theorem 5.1 on white noise intensity ($\sigma _{1}^{2}<7.24\times 10^{-3}$, $\sigma _{2}^{2}<7.24\times 10^{-3}$, $\sigma _{3}^{2}<0.41429$, $\sigma _{4}^{2}<0.11429$, $\sigma _{5}^{2}<4.74900\times 10^{-5}$, $\sigma _{6}^{2}<1.53087\times 10^{-4}$).

**Figure 1:**
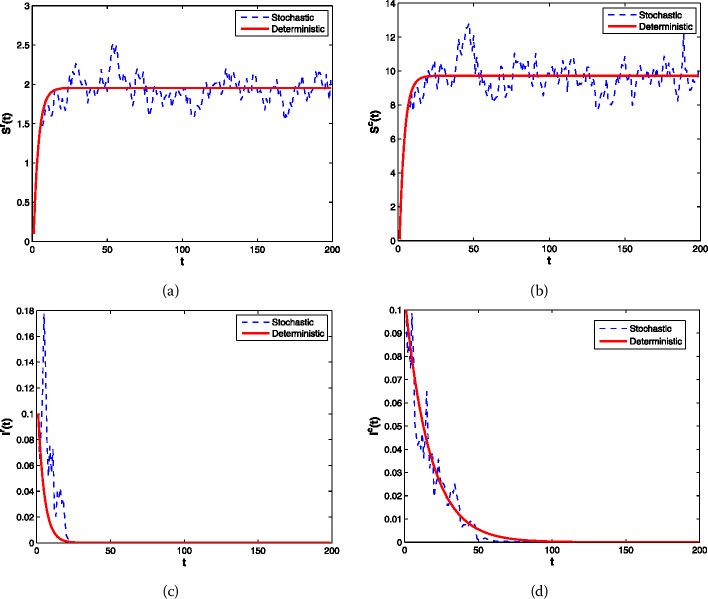
Trajectories (**a**, **b**, **c**, **d**) of the deterministic model (2) and the stochastic model (3)

**Figure 2:**
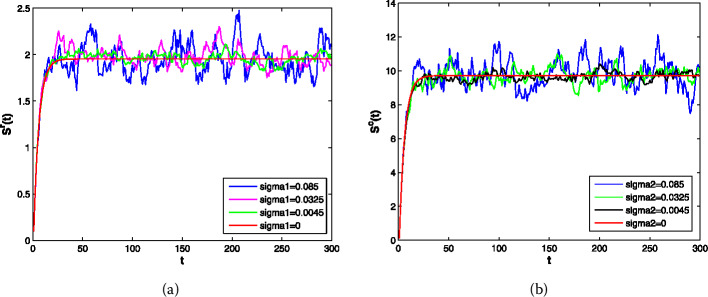
Trajectories (**a**, **b**) of the stochastic model (3) for different white noise intensity

(i) Four variables $S^{r}$, $S^{c}$, $I^{r}$, $I^{c}$ are picked. In Fig. 1(a, b, c, d), it represents the oscillation of the random model (3) near the disease-free equilibrium point $E_{0}=( 1.95417 , 9.72222 , 0 , 0 , 68.39591 , 340.27814)$. The result obtained by the numerical simulation is in good agreement with Theorem 5.1. Figure 1(c, d) indicates that the outbreak of disease can be suppressed with moderate intensity of noise, and it can be seen that the probability of extinction of this disease is one with the increase of time.

(ii) In Fig. 2(a and b), the intensity of other noise is kept constant, while $\sigma _{1}$ and $\sigma _{2}$ are changed respectively; this shows that the fluctuation of the random model becomes more and more obvious with the increase in the intensity of noise. That is to say, the intensity of the disturbance is proportional to the intensity of noise.

## Conclusions

Environmental noise can be described to have a significant effect on the advancement of an epidemic. For this study, we present the dynamics of a stochastic two-group SVIR model under the noises of environment. We suppose that the stochastic perturbation is of a white noise sort that disturbs the natural death rate *μ*.

Compared with the deterministic model (2), we find that the intensity of the noise level plays a critical role. Therefore, our main results are summarized as follows.

The existence and uniqueness of positive solution is proved by using stopping time theory and the Lyapunov analysis method. We all know that controlling the spread of disease is a very important task in epidemiology, but it requires some theoretical foundation. So we studied the property of the systematic solution and the asymptotic behavior of that solution around the corresponding deterministic model disease-free equilibrium point. We obtain Theorem 4.1, and the solution of model (3) is oscillated randomly around the disease-free equilibrium point $E_{0}$. From Theorem 5.1, if $R_{0}<1$ under the small noise intensity case, that is, $\sigma _{1}^{2}<\frac{1}{1+p} ( \frac{p\mu (\mu +\theta ^{r})}{\mu +2\theta ^{r}}-(2\mu +\nu ^{r}) )$, $\sigma _{2}^{2}<\frac{1}{1+q} ( \frac{q\mu (\mu +\theta ^{c})}{\mu +2\theta ^{c}}-(2\mu +\nu ^{c}) )$, $\sigma _{5}^{2}<\frac{\theta ^{r}}{p\mu +\theta ^{r}} ( \frac{p\mu ^{3}}{2\theta ^{r}(\theta ^{r}+\mu )}-(2\mu +\nu ^{r}) )$, $\sigma _{6}^{2}<\frac{\theta ^{c}}{q\mu +\theta ^{c}} ( \frac{q\mu ^{3}}{2\theta ^{c}(\theta ^{c}+\mu )}-(2\mu +\nu ^{c}) )$, $\sigma _{3}^{2}<(\mu +\nu ^{r})$, $\sigma _{4}^{2}<(\mu + \nu ^{c})$, the solution $(\frac{\Lambda ^{r}}{\mu +\theta ^{r}} , \frac{\Lambda ^{c}}{\mu +\theta ^{c}} , 0 , 0 , \frac{\theta ^{r}\Lambda ^{r}}{\mu ^{2}+\mu \theta ^{r}} , \frac{\theta ^{c}\Lambda ^{c}}{\mu ^{2}+\mu \theta ^{c}} )$ is found to be stochastically asymptotically stable. This reveals that the stochastic model (3) has disease extinction with probability one. Particularly, the noise intensity is zero when the models become the deterministic model (2), thereby the disease is extinct too. Finally, the results are verified by a numerical simulation.

In comparison with our model, the model established by Zhang and Yang [29] is a deterministic model without considering the influence of environmental factors. Thus, the model established by us is more consistent with the real infectious disease dynamic process. Based on this, our work seems more precise and further enriches their results.

Nevertheless, only the asymptotic behavior of the model is discussed in this paper. In our upcoming work, we will further discuss other properties of the model, such as ergodic property and the existence of an invariant distribution. And we would build some model with time delay, age composition, and control item.

## Data Availability

Not applicable.
